# Global burden of prostate cancer (1990–2021) from Brazil: trends in disability-adjusted life years, incidence, mortality, and prevalence

**DOI:** 10.1007/s10552-026-02232-2

**Published:** 2026-08-19

**Authors:** Gabriela Daiprai, Bruna Cristina Jordon Togni, Maria Noêmia Souza de Alcântara, Joana Ecco, Ágatha Kniphoff da Cruz, Fernanda Majolo, Guilherme Liberato da Silva

**Affiliations:** 1 Graduate Program in Medical Sciences, University of Taquari Valley - Univates, Av. Avelino Talini, 171 - Universitário, Lajeado, Rio Grande Do Sul Brazil; 2 Graduate Program in Biotechnology, University of Taquari Valley - Univates, Av. Avelino Talini, 171 - Universitário, Lajeado, Rio Grande Do Sul Brazil; 3 Medical Sciences Center, University of Taquari Valley - Univates, Av. Avelino Talini, 171 - Universitário, Lajeado, Rio Grande Do Sul 95914-014 Brazil

**Keywords:** AAPC, Health disparities, DALY, Macroregions, Oncology

## Abstract

**Background:**

Prostate cancer remains a significant public health challenge globally, with marked regional disparities in Brazil linked to demographic, socioeconomic, and healthcare access factors. This study analyzed trends in disability-adjusted life years (DALYs), incidence, mortality, and prevalence of prostate cancer across Brazilian macroregions (1990–2021) using data from the Global Burden of Disease (GBD) database. Joinpoint regression was employed to identify temporal trends and average annual percentage changes (AAPCs), stratified by age and region.

**Methods:**

Data were extracted from GBD 2021, encompassing national and regional estimates. Statistical analyses included age-standardized rates, AAPC calculations, and pairwise comparisons to assess parallelism in trends.

**Results:**

Results revealed substantial regional disparities: Southern and Southeastern regions exhibited the highest DALYs, incidence, and prevalence, patterns that are consistent with better diagnostic infrastructure and aging populations. In contrast, Northern regions showed lower rates but the steepest AAPC increases (e.g., incidence: + 2.59% annually in age group 70–74), suggestive of improving yet insufficient healthcare access. Mortality declined in the South and Southeast (AAPC up to −0.70% in age group 70–74) due to therapeutic advances but rose in the Northeast (AAPC up to + 0.76% in age group 65–69). Prevalence surged nationwide, peaking in older age groups (e.g., 2,619.50 cases/100,000 men in Southern 70–74 age group), indicating prolonged survival. Joinpoint analyses identified non-linear trends, with incidence stabilizing post-2010 following shifts in screening guidelines.

**Conclusion:**

These findings underscore the need for region-specific policies to address inequities in cancer care, enhance early diagnosis, and optimize resource allocation. Strengthening healthcare infrastructure in underserved regions and targeted screening for younger high-risk populations are critical to mitigating Brazil’s prostate cancer burden.

**Supplementary Information:**

The online version contains supplementary material available at https://doi.org/10.1007/s10552-026-02232-2.

## Introduction

Prostate cancer is a neoplasm predominantly associated with aging, being most frequent in individuals over 65 years of age. It represents a major public health challenge worldwide and is considered the 4th most common type of cancer globally. In Brazil, the National Cancer Institute (INCA) highlights that prostate cancer is the second most frequent cancer among men, second only to non-melanoma skin cancer. Among the Brazilian male population, prostate cancer is the second leading cause of cancer death, surpassed only by lung cancer [1]. The main recognized risk factors for its etiology are senescence, genetic predisposition, sedentary lifestyle, African ancestry, and the chronic use of certain medications, such as anabolic–androgenic steroids and testosterone replacement therapy, which have been associated with accelerated growth of latent prostate tumors [2]. Prostate cancer progression is slow, particularly in early stages and low-grade tumors. According to a Swedish population-based cohort study that followed 223 men with localized, untreated prostate cancer for over 30 years, the majority of tumors followed an indolent course during the first 15 years after diagnosis. However, after this period, there was a significant increase in disease progression and prostate cancer-specific mortality [2, 3].

An estimated 71,730 new prostate cancer cases are projected in Brazil for the 2023–2025 triennium, corresponding to an incidence rate of 67.86 per 100,000 men. Mortality data from the Brazilian Mortality Information System (SIM) indicate that prostate cancer deaths have increased steadily over the past two decades, from approximately 15,983 deaths in 2019 to 17,093 deaths in 2023, an average of 47 deaths per day. This rising mortality trend may be linked to population aging and potential diagnostic/treatment delays during the COVID-19 pandemic [1, 4]. Prostate cancer incidence varies widely worldwide, influenced by factors like population life expectancy and local diagnostic methods. Data collection practices also significantly impact reported incidence rates [5]. Therefore, it becomes essential to understand prostate cancer trends and accurately assess its burden at both national and subnational levels, thereby informing more effective public health policies particularly given Brazil’s vast territorial expanse and significant regional disparities.

Brazil’s South and Southeast regions account for approximately 70% of new prostate cancer cases, with incidence rates of 77.89 (Southeast) and 57.23 (South) per 100,000 men (INCA, 2023). This disparity reflects higher population density, better diagnostic infrastructure, and longer life expectancy in these regions [6]. In contrast, the North has the lowest incidence (28.40/100,000) but shows the highest annual growth (7.2%), suggesting recent—though insufficient—gains in diagnostic coverage, as only 30% of its municipalities have urologists [6]. Oncology services are also unevenly distributed, while the Southeast concentrates most high-complexity facilities, the North relies heavily on travel to capitals like Belém and Manaus, where radiotherapy demand exceeds capacity by 78% [6].

Brazilian Prostate Cancer Guidelines have undergone continuous revisions over the past 15 years. Between 2004 and 2010, mass screening expansion was recommended by the American Cancer Society using PSA (Prostate-Specific Antigen) testing and digital rectal examination. This practice resulted in overdiagnosis and overtreatment, where tumors unlikely to ever cause symptoms or death were aggressively treated [7, 8]. In 2012, Brazil transitioned from near-automatic PSA screening to a more selective approach following publication of the PLCO Trial (Prostate, Lung, Colorectal, and Ovarian Cancer Screening Trial, USA) and ERSPC Trial (European Randomized Study of Screening for Prostate Cancer) findings [9, 10]. A pivotal 2018 update by the USPSTF (U.S. Preventive Services Task Force) subsequently influenced international guidelines, including Brazil’s. This framework emphasized shared decision-making, where patients are informed of benefits and risks prior to screening [11]. Within this context, disability-adjusted life years (DALYs) emerge as a critical metric. By integrating morbidity and mortality data, DALYs provide a comprehensive disease burden assessment. They quantify both years of life lost to premature mortality and years lived with disability, enabling precise cross-population comparisons of cancer impact. This facilitates evidence-based resource allocation and development of prevention/treatment strategies [12].

The Global Burden of Diseases, Injuries, and Risk Factors Study (GBD) provides the foundational epidemiological estimates for our analysis, offering comprehensive and standardized data on disease burden across 204 countries and territories, including subnational estimates for Brazil [17]. The GBD 2021 study, in particular, synthesized vast amounts of data to produce global and national estimates for 371 diseases, highlighting major shifts in burden due to the COVID-19 pandemic and ongoing epidemiological transitions [17]. While GBD furnishes these indispensable national-level estimates, its primary focus is on global and regional comparisons. Our study builds upon this foundation by conducting a deep-dive analysis specifically tailored to Brazil’s five macroregions.

The present study is guided by the following hypotheses: (1) Significant variation exists across macroregions and between age groups regarding prostate cancer incidence, prevalence, DALY (disability-adjusted life years), and mortality rates, enabling analysis of disparities in healthcare access, socioeconomic factors, and demographic characteristics; (2) Older age will be directly associated with increased incidence, prevalence, and mortality rates, with population peaks observed above age 65, whereas DALY will demonstrate a higher burden in younger age groups (e.g., 50–64 years) due to the relative impact of years lost to early disability or premature death; 3) Non-linear temporal trends: Over the study period (1990–2021), an initial increase in incidence (until year 2010) will be observed, associated with expanded PSA screening, followed by stabilization or decline due to changes in clinical guidelines; progressive reduction in mortality and DALY rates, especially post-2000, driven by therapeutic advances (e.g., targeted radiotherapy, hormone therapy); increased prevalence, indicating improved patient survival, with regional variations reflecting health policy implementation.

Consequently, this study aims to determine prostate cancer incidence, prevalence, mortality, and DALY trends over the past three decades (1990–2021) using the Global Burden of Disease (GBD) platform for Brazil and its macroregions.

## Methods

This is a descriptive-analytical study examining the epidemiological burden of prostate cancer in Brazil and its macroregions from 1990 to 2021. Data were extracted from the visualization tools of the Institute for Health Metrics and Evaluation [13], which hosts results from the Global Burden of Disease Study [14–17]—a comprehensive database measuring the impact of 371 diseases, 88 risk factors, and injuries across 204 countries and territories.

For our study, we obtained estimates of incidence, prevalence, mortality, and DALY (Disability-Adjusted Life Years) for prostate cancer in Brazil and its macroregions. The primary source for cause-of-death data in Brazil is the Mortality Information System (*Sistema de Informação de Mortalidade—SIM—Portuguese version*). Within the GBD framework, the so-called “garbage codes”—deaths assigned to causes that cannot be underlying causes of death (or are implausible for other reasons)—are redistributed to plausible underlying causes using a variety of methodological approaches. After redistributing these garbage-coded deaths, the corrected mortality database serves as an input for the *Cause of Death Ensemble modeling* (CODEm) tool to estimate cause-specific mortality patterns.

For prevalence, incidence, and years lived with disability (YLDs) estimates, the Global Burden of Disease (GBD) study employs the *DisMod-MR 2.123* tool, which utilizes Bayesian meta-regression. In Brazil, source data for non-fatal health outcomes and covariates used to compute these estimates derive primarily from databases, survey data, other epidemiological studies, registries, and disease-specific databases. DALY (Disability-Adjusted Life Year) constitutes an effective metric for quantifying disease burden in populations, incorporating both fatal and non-fatal health outcomes. This measure is calculated by combining years of life lost (YLL) due to premature mortality with years lived with disability (YLD), where one DALY represents the loss of one equivalent year of ideal healthy life. Understanding prostate cancer DALY, along with mortality, prevalence, and incidence rates, is essential as it provides a comprehensive assessment of the true burden of prostate cancer across the populations [18, 19].

### Data analysis

The methodology employed in the Global Burden of Disease (GBD) Study 2021 has been extensively detailed in prior publications [14, 15]. Data are reported per 100,000 male population. For statistical analyses, two-tailed tests were adopted, with significance defined by *p* values below 0.05. Temporal trends in the burden of prostate cancer were assessed using joinpoint regression analysis via the Joinpoint Regression Program [20–22], developed by the Surveillance Research Program of the U.S. National Cancer Institute. This tool identifies temporal variations in the data and constructs simplified models by connecting linear segments on a logarithmic scale.

All rates (incidence, prevalence, mortality, and DALYs) are reported per 100,000 men per year. The denominators used to calculate these rates are the population estimates for Brazilian men, derived from the GBD 2021 study. These GBD population estimates are based on multiple sources, including censuses (1991, 2000, 2010), inter-censal surveys, and vital registration data, standardized and harmonized across all years (1990–2021) using the GBD comparative population assessment framework. Age-standardized rates, when calculated for supplementary analyses, were standardized to the GBD world population standard. The sociodemographic context is discussed qualitatively by referencing established literature on the Human Development Index (HDI) and healthcare infrastructure across macroregions, but these were not included as quantitative covariates in the regression models. DALYs (Disability-Adjusted Life Years) are calculated as the sum of Years of Life Lost due to premature mortality (YLLs) and Years Lived with Disability (YLDs): DALY = YLL + YLD. One DALY represents the loss of one year of healthy life. For prostate cancer, the GBD study estimates YLLs from cause-specific mortality data and YLDs from prevalence estimates multiplied by disability weights for the health states associated with prostate cancer and its sequelae.

To quantify trends, the average annual percentage change (AAPC) was calculated [23]. This indicator synthesizes the annual percentage changes identified in the analysis, geometrically weighted by the duration of each time interval. Ninety-five percent confidence intervals (95% CIs) were derived from linear regression models. An upward trend was considered when the AAPC and its 95% CI exceeded zero, and a downward trend when both were below zero. If the 95% CI includes zero, the age-standardized rate (ASR) was deemed stable over the analyzed period [24]. The AAPC is a summary measure that represents the average annual percentage change in the rate over the entire study period (1990–2021). Unlike the APC (Annual Percent Change), which describes the slope of each individual segment between joinpoints, the AAPC is calculated as a geometrically weighted average of the APCs, taking into account the duration of each segment. Thus, the AAPC synthesizes the overall trend for the period into a single value, even when multiple changes in direction (joinpoints) are identified.

The standard error (SE) was estimated using the formula: SE = (upper limit − lower limit)/(1.96 × 2), where the limits correspond to the boundaries of the CI obtained from GBD data. The final model selection was based on the Bayesian Information Criterion (BIC). These data were stratified by age (40–44, every 5-year age group up to 95 years, and 95 years and older), calendar year (1990–2021), macroregions (South, Southeast, Central-West, North, Northeast).

Pairwise comparison in joinpoint regression analysis evaluates whether temporal trends between two groups (e.g., age group) are statistically similar in structure. This method fits segmented regression models to each group, identifying joinpoints (points where the trend slope changes significantly). To assess parallelism, the analysis tests two hypotheses: 1—Equal number of joinpoints: Whether both models share the same number of inflection points. 2—Parallel trends: Whether the slopes of corresponding segments (between joinpoints) are statistically equivalent. A permutation test (4500 permutations) calculates the *p* value to determine if observed differences exceed random variation. If the *p* value exceeds the predefined significance level (α = 0.05), the null hypothesis of parallelism is not rejected, implying trends follow comparable patterns. Conversely, a *p* value ≤ α indicates divergent trends (“Not Parallel”). This approach allows robust identification of shared or distinct epidemiological behaviors across groups over time.

### Ethics statement

The use of anonymized, publicly available epidemiologic data did not require ethical approval, and patient informed consent forms were not necessary when accessing and downloading the data from the database.

## Results

Descriptive statistics comparing regions, stratified by age groups in the 55–59 age group, moderate regional variation is observed across health indicators. The Southeast region recorded the highest mean DALY (548.64 ± 68.28), followed by the Northeast (538.72 ± 69.76) and Central-West (523.98 ± 69.62). The lowest value was registered in the North (481.97 ± 61.26). The highest incidence was also in the South (60.44 ± 6.82), while the North had the lowest (45.95 ± 9.73). For mortality, the Southeast leads with (15.32 ± 2.00), and the North again shows the lowest value (13.57 ± 1.69). Regarding prevalence, the South stands out (510.88 ± 64.75), with the lowest value observed in the North (384.44 ± 86.66).

In the 60–64 age group, a substantial increase is observed across all indicators. The highest mean DALY rate was recorded in the South (1251.04 ± 166.71), followed by the Southeast (1245.97 ± 151.41). The lowest value remained in the North (1076.99 ± 126.55). Incidence maintained a similar pattern, with the South again showing the highest value (139.70 ± 14.91) and the North the lowest (100.87 ± 21.49). Mortality rates ranged from 35.26 ± 3.99 (North) to 40.45 ± 5.18 (Southeast). Prevalence was highest in the South (1135.55 ± 138.46) and lowest in the North (812.64 ± 190.66).

In the 65–69 age group, values continue to rise. The South region again records the highest DALY (2214.43 ± 264.58), followed by the Southeast (2124.57 ± 277.75) and Central-West (2114.32 ± 251.73). The North maintains the lowest Figs. (1854.64 ± 218.81). Incidence follows the same trend: highest in the South (247.43 ± 28.30) and lowest in the North (175.87 ± 35.78). Mortality ranges from 72.11 ± 8.23 (North) to 84.90 ± 10.62 (South). For prevalence, the South again stands out (1904.76 ± 263.17), while the North continues with the lowest values (1340.92 ± 312.22).

Values reach their highest elevation in the 70–74 age group. The highest mean DALY remains in the South (3590.60 ± 388.05), while the North has the lowest value (2993.86 ± 383.49). Incidence ranges from 272.32 ± 53.75 (North) to 377.67 ± 42.45 (South). The highest mortality was again observed in the South (168.20 ± 18.79), with the North recording 142.03 ± 17.76. Prevalence follows a similar pattern: highest in the South (2619.50 ± 394.85) and lowest in the North (1846.18 ± 442.55).

**Fig. 1:**
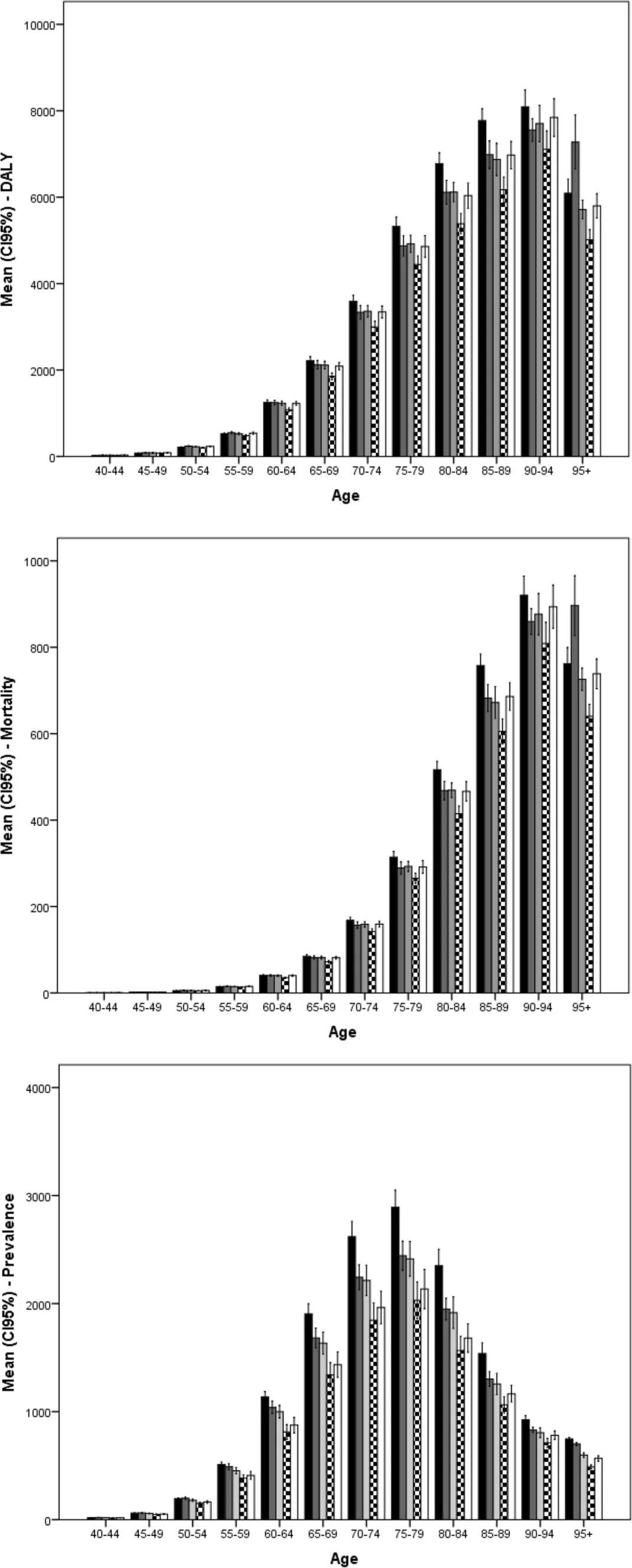

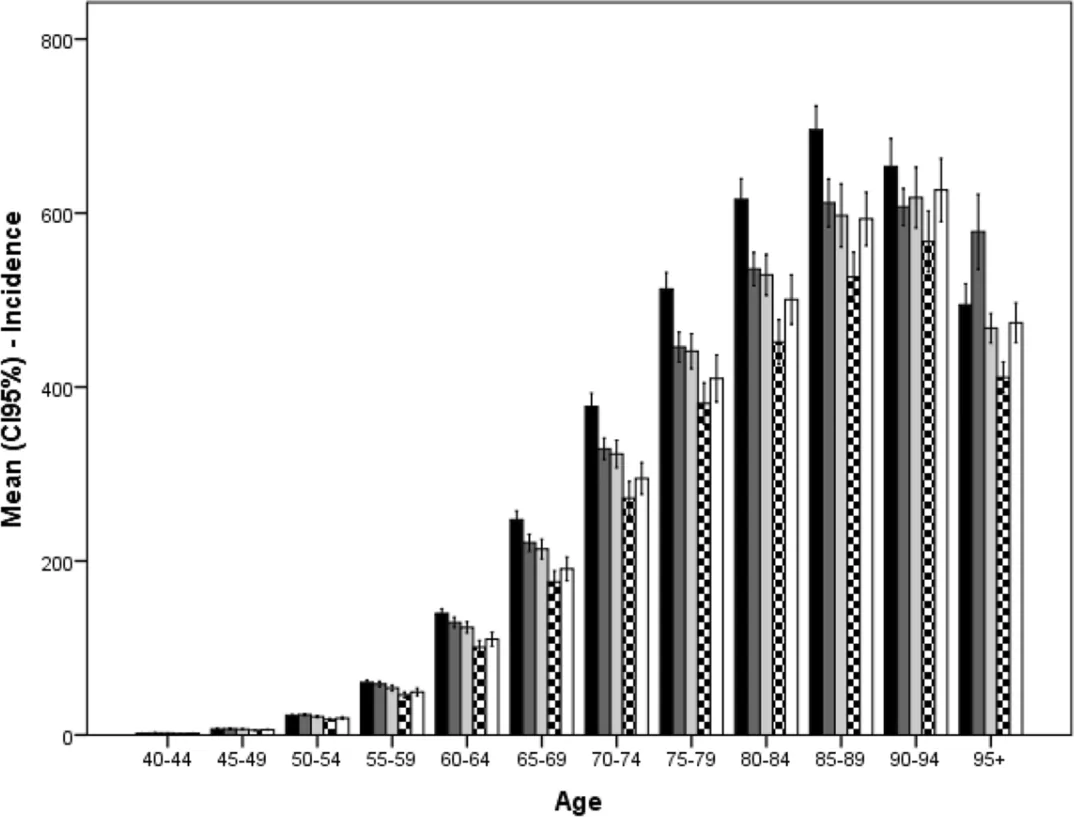
Age-specific trends in disability-adjusted life years (DALY), mortality, incidence, and prevalence rates. Data are presented as mean values with 95% confidence intervals (C95%) across different age groups

For the 75–79 age group, the South region reports the highest DALY value (5322.36 ± 603.48), followed by the Southeast (4872.43 ± 648.95) and Central-West (4920.42 ± 556.89). The lowest, though still high, average was observed in the North (4442.18 ± 553.38). Incidence rates range from 381.27 ± 65.64 in the North to 512.50 ± 53.35 in the South. Mortality was also highest in the South (314.45 ± 36.35) and lowest in the North (265.47 ± 32.36). Regarding prevalence, the South again leads (2890.94 ± 445.68), while the North showed the lowest value (2030.16 ± 475.55) (Fig. 1).

### AAPC

#### Mortality

**Fig. 2:**
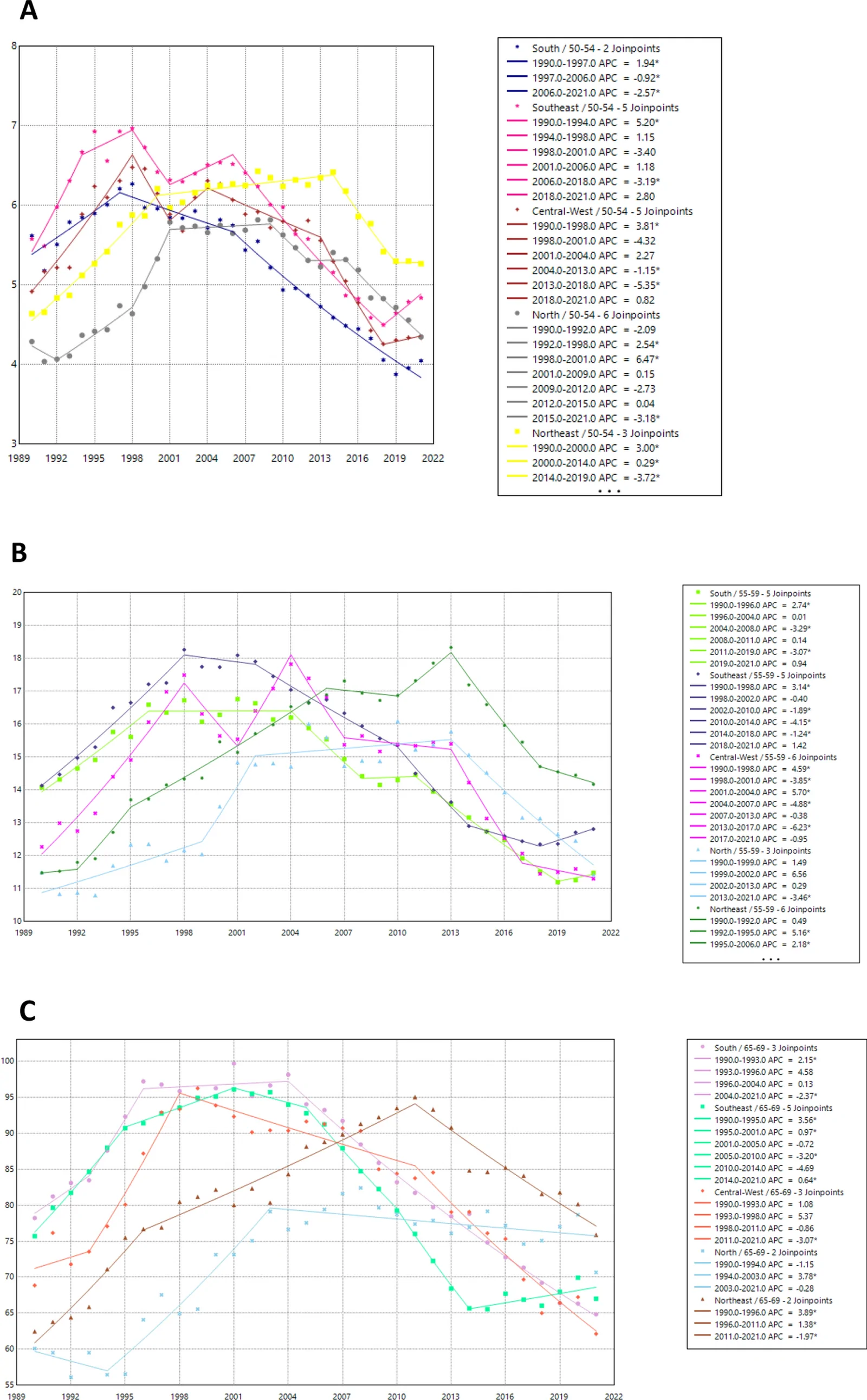
Joinpoint regression analysis of age-specific mortality trends across Brazilian regions (South, Southeast, Central-West, North, Northeast) from 1990 to 2021. The x-axis represents the calendar years (1990–2021), and the y-axis represents the mortality rate per 100,000 men. Panels A–C represent age groups in ascending order: 50–54 (**A**), 55–59 (**B**), 65–69 (**C**). Annual percentage change (APC) values with corresponding joinpoints are illustrated for each region, highlighting temporal shifts in mortality rates. *Indicates that the APC is statistically significant (*p* < 0.05)

Regarding AAPC (Average Annual Percentage Change) in mortality, the 40–44 and 45–49 age groups showed no significant change during the 1990–2021 period across all macroregions. However, significant declines were observed in the South region for ages 50–54 (− 1.08%/year), 55–59 (− 0.63%/year), and 65–69 (− 0.64%/year). Conversely, these same age groups exhibited significant mortality increases in the Northeast: + 0.47%/year (50–54), + 0.69%/year (55–59), and + 0.76%/year (65–69) (Fig. 2).

The Southeast region demonstrated notable annual reductions: −0.64% (70–74), −0.51% (75–79), and −0.70% (80–84). It should be emphasized that the oldest cohorts (85–89 and 90–94) showed significant mortality increases in both the South (+ 0.64% and + 1.75%/year) and Northeast (+ 0.81% and + 1.15%/year) (Table S1).

When considering a broader age group (50–74 years), mortality shows a trend of annual increase in the North and Northeast regions, whereas in the Central-West, Southeast, and South regions, a reduction is observed (Figure S1).

#### Prevalence

**Fig. 3:**
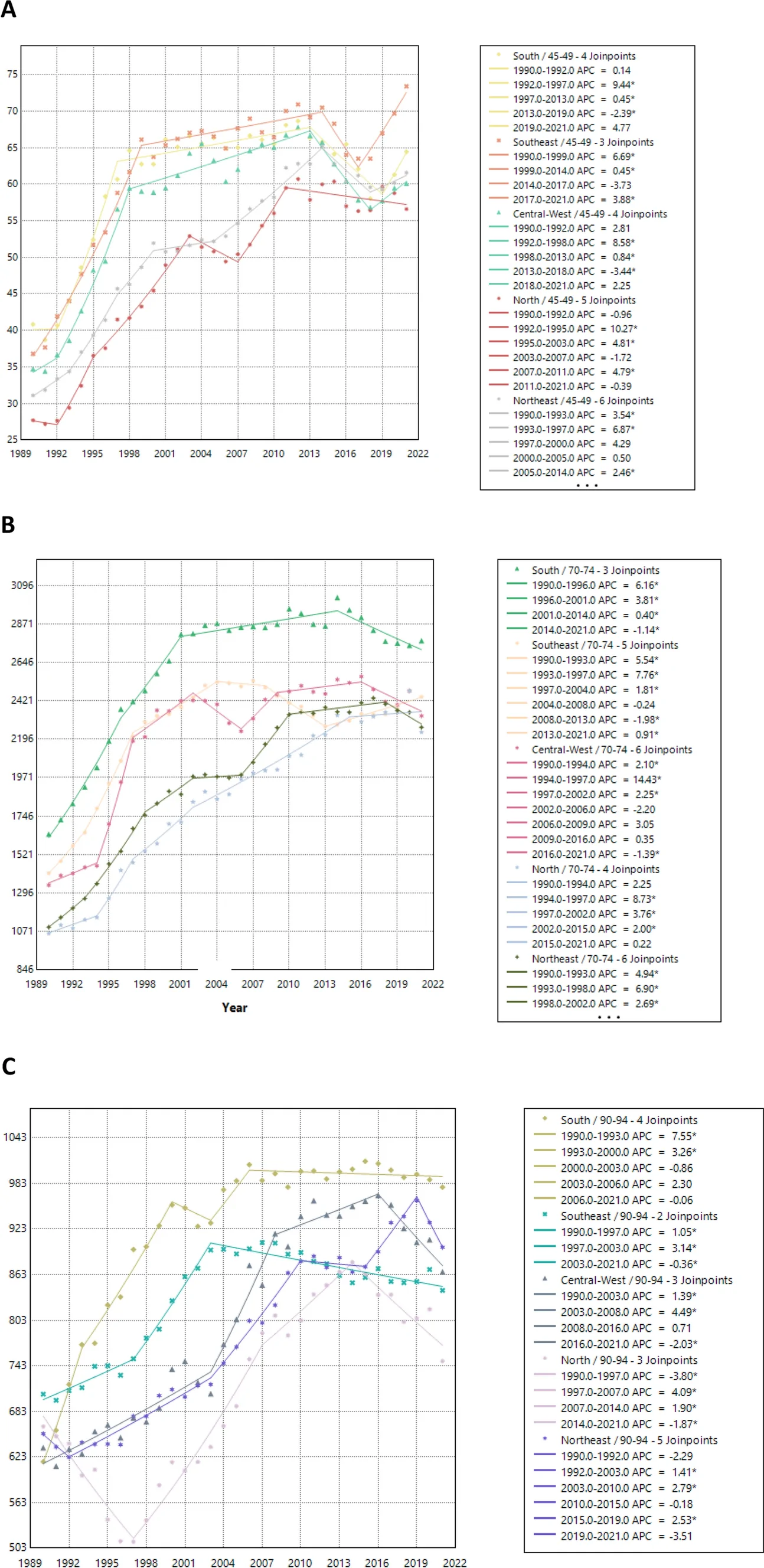
Joinpoint regression analysis of age-specific prevalence trends across Brazilian regions (South, Southeast, Central-West, North, Northeast) from 1990 to 2021. The x-axis represents the calendar years (1990–2021), and the y-axis represents the prevalence rate per 100,000 men. Panels A–C represent age groups in ascending order: 45–49 (**A**), 70–74 (**B**), 90–94 (**C**). Annual percentage change (APC) values with corresponding joinpoints are illustrated for each region, highlighting temporal shifts in prevalence rates. *Indicates that the APC is statistically significant (*p* < 0.05)

Regarding the AAPCs (Average Annual Percentage Changes) for disease prevalence, we observe that age groups from 40 to 89 years experienced a significant increase in cases across all macroregions of Brazil. When examining the highest increases by age group, it can be concluded that the Central-West region had the highest AAPC for the 80–84 (2.64%) and 85–89 (2.19%) age groups. However, the Northeast region exhibited the highest AAPC percentages in nearly all studied age groups: 40–44 (2.1%), 50–54 (2.55%), 55–59 (2.85%), 60–64 (2.21%), 65–69 (2.74%), 75–79 (2.57%), and 95 + (0.68%). This was followed by the North region, which showed the highest AAPCs for the 45–49 (2.37%) and 70–74 (2.59%) age groups (Fig. 3). The South region showed the highest AAPC only in the 90–94 (1.55%) age group (Fig. 3; Table S2).

Overall, when assessing the 50–74 age group, the prevalence increases in all macroregions, with the highest rate of increase in the Northeast and the lowest in the South (Figure S2).

### DALYs

**Fig. 4:**
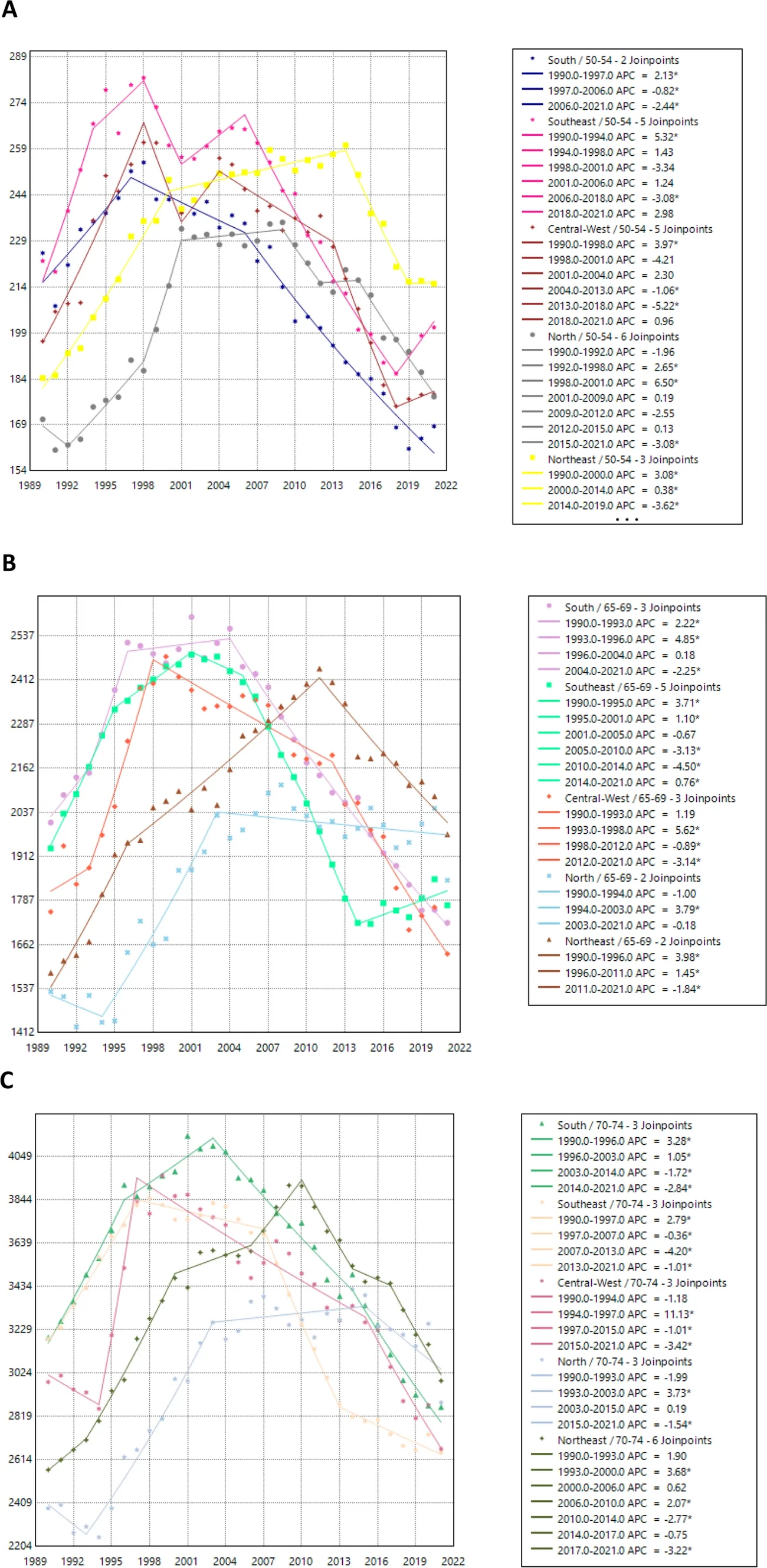
Joinpoint regression analysis of age-specific DALYs trends across Brazilian regions (South, Southeast, Central-West, North, Northeast) from 1990 to 2021. The x-axis represents the calendar years (1990–2021), and the y-axis represents the DALYs rate per 100,000 men. Panels A–C represent age groups in ascending order: 50–54 (**A**), 65–69 (**B**), 70–74 (**C**). Annual percentage change (APC) values with corresponding joinpoints are illustrated for each region, highlighting temporal shifts in DALYs rates. *Indicates that the APC is statistically significant (*p* < 0.05)

Concerning the AAPC for DALYs, the 40–44 and 45–49 age groups showed no significant change over the time period (1990–2021) in any macroregion. However, the Northeast region exhibited significantly higher AAPC increases for DALYs in nearly all studied age groups: 50–54 (0.56%), 55–59 (0.79%), 60–64 (0.73%), 65–69 (0.85%), 70–74 (0.52%), 75–79 (0.78%), 80–84 (0.43%), 85–89 (0.82%), 90–94 (1.15%), and 95 + (0.82%). It is noteworthy that the South experienced significant declines in AAPC for the 50–54 (−0.95%), 65–69 (−0.53%), and 70–74 (−0.4%) age groups, while the Southeast region recorded significant decreases in AAPC percentages for the 60–64 (−0.37%), 70–74 (−0.58%), 75–79 (−0.43%), 80–84 (−0.65%), and 95 + (−1.76%) age groups (Fig. 4; Table S3). The burden of disease, measured in DALYs, shows an annual increase in the North and Northeast when considering the 50–74 age group, while a slight stability is observed in the Central-West and a reduction in the Southeast and South (Figure S3).

#### Incidence

**Fig. 5:**
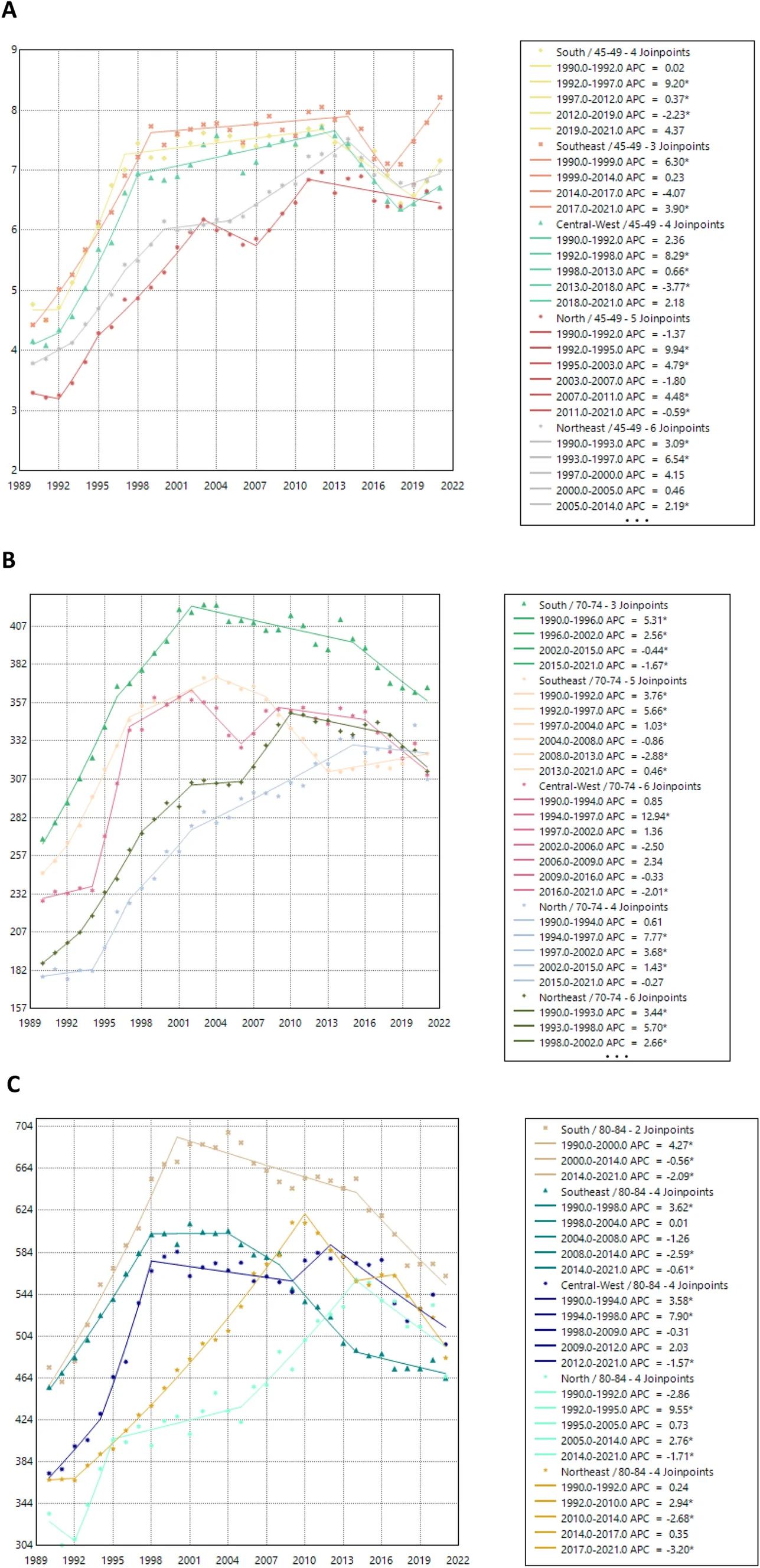
Joinpoint regression analysis of age-specific incidence trends across Brazilian regions (South, Southeast, Central-West, North, Northeast) from 1990 to 2021. The x-axis represents the calendar years (1990–2021), and the y-axis represents the incidence rate per 100,000 men. Panels A–C represent age groups in ascending order: 45–49 (**A**), 70–74 (**B**), 80–84 (**C**). Annual percentage change (APC) values with corresponding joinpoints are illustrated for each region, highlighting temporal shifts in incidence rates. *Indicates that the APC is statistically significant (*p* < 0.05)

About AAPCs for prostate cancer incidence, it was observed that age groups from 40 to 74 years showed significant increases in AAPCs across all Brazilian macroregions. When analyzing the highest increases by age group, it can be concluded that the Northeast region recorded the highest AAPC percentages for incidence in nearly all studied age brackets: 40–44 (1.91%), 50–54 (2.3%), 55–59 (2.57%), 60–64 (2.24%), 65–69 (2.26%), 75–79 (1.60%), 85–89 (1.15%), and 95 + (0.86%). The North region demonstrated the highest AAPC percentages in the 45–49 (2.2%), 70–74 (1.94%), and 80–84 (1.33%) age groups (Fig. 5).

The only significant antagonistic result was observed in the Southeast region for the 95 + age group, which experienced an AAPC decline of 1.48%. (Table S4).

When analyzing incidence in the broader age group of 50 to 74 years, an increase was observed across all regions, with a more pronounced rise in the North and Northeast and a less marked increase in the South (Figure S4).

### Pairwise comparisons—age groups in Brazil

The 40–44 and 45–49 age groups exhibited parallel temporal trends (*p* = 0.11) about incidence rates, with three significant change points (joinpoints) in each curve. The 55–59 age group showed parallelism with the 45–49 group (4 joinpoints; *s* = 0.26) and with the 50–54 group (3 joinpoints; *p* = 0.057). Comparison of prostate cancer incidence trends between the 50–54 and 55–59 age groups revealed parallel trajectories, indicating structurally similar temporal patterns. The AAPC for both groups was 1.88% per year (95% CI 1.62–2.13; *p* < 0.05), suggesting a consistent and statistically significant increase in incidence from 1990 to 2021.

**Fig. 6:**
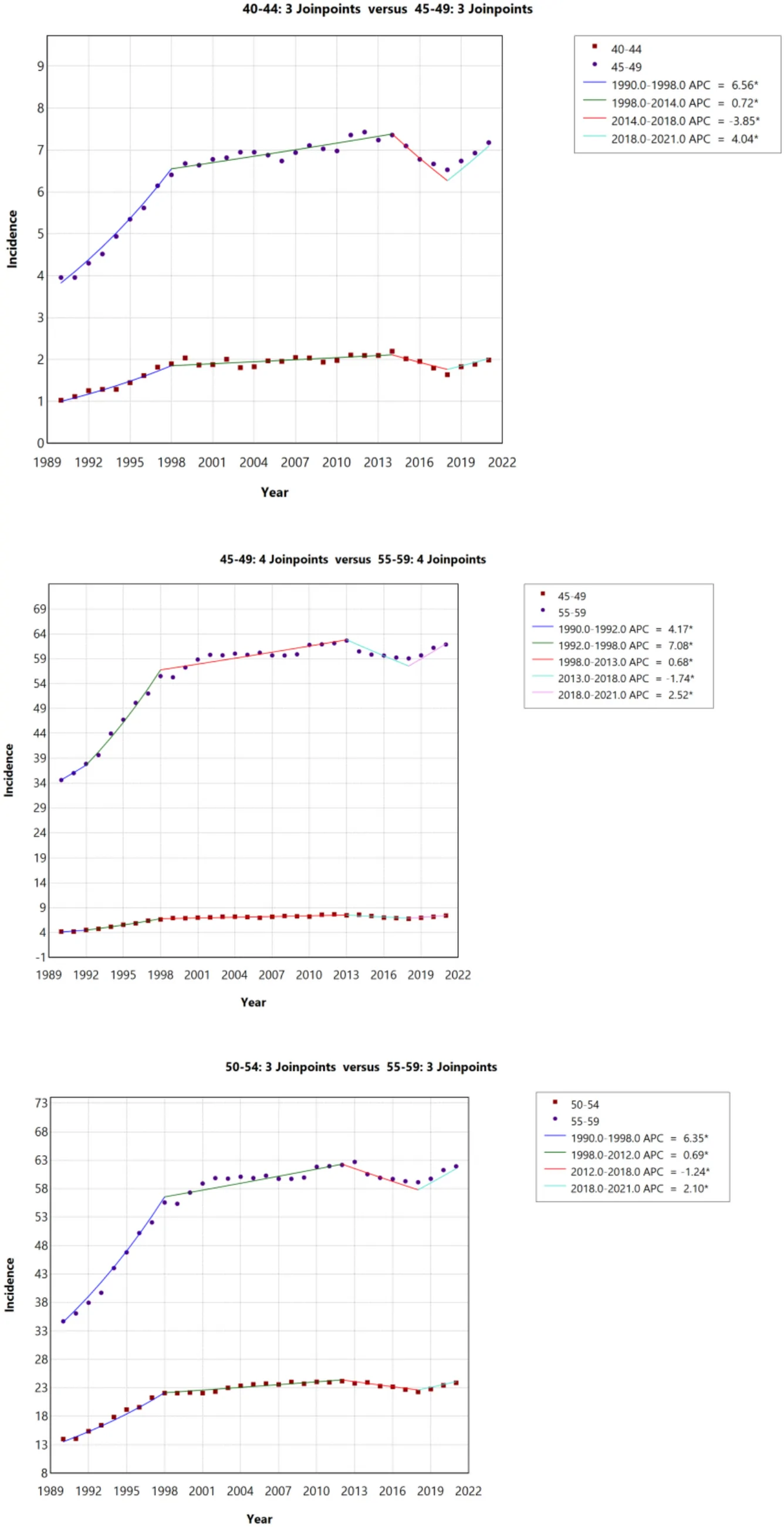
Pairwise comparison of prostate cancer incidence trends (age groups: 40–44, 45–49, and 55–59 years) demonstrating parallel trajectories from 1990 to 2021. Joinpoint regression analysis identifies significant inflection points (joinpoints) and corresponding annual percent change (APC) values for each trend segment. The parallel patterns suggest similar temporal dynamics across these age groups. Data derived from joinpoint regression (*Indicates that the Annual Percent Change (APC) is significantly different = *p* < 0.05)

Most other pairwise age group comparisons showed non-parallelism (*p* < 0.05), evidencing distinct temporal changes over time. For example, comparative analysis of prostate cancer incidence trends between the 55–59 and 65–69 age groups revealed divergent trajectories (*p* = 0.0002), indicating dissimilar temporal patterns. However, the AAPC comparison was non-significant, suggesting no significant difference in the average annual percent change between these groups over the entire period (Fig. 6).

**Fig. 7:**
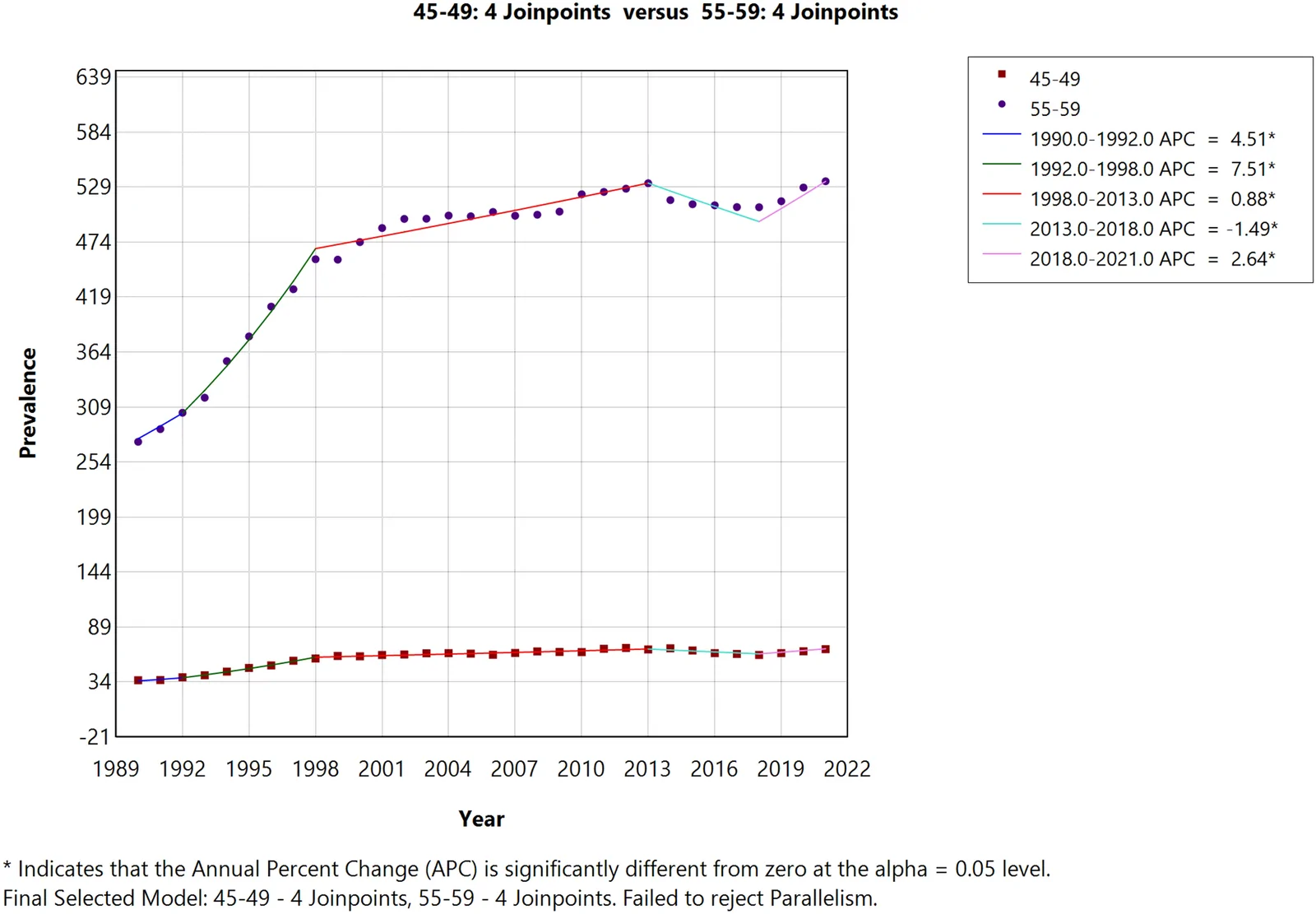
Pairwise comparison of prostate cancer prevalence trends (age groups: 45–49 and 55–59 years) demonstrating parallel trajectories from 1990 to 2021. Joinpoint regression analysis identifies significant inflection points (joinpoints) and corresponding annual percent change (APC) values for each trend segment. The parallel patterns suggest similar temporal dynamics across these age groups. Data derived from joinpoint regression (*Indicates that the Annual Percent Change (APC) is significantly different = *p* < 0.05)

Concerning the prevalence rates, the 45–49 and 55–59 age groups also demonstrated parallelism (4 joinpoints; *p* = 0.34), with an AAPC for both groups suggesting an increase of 2.14% per year (95% CI: 1.81–2.47; *p* < 0.001) during the study period. All other pairwise age group combinations showed no parallelism (Fig. 7).

**Fig. 8:**
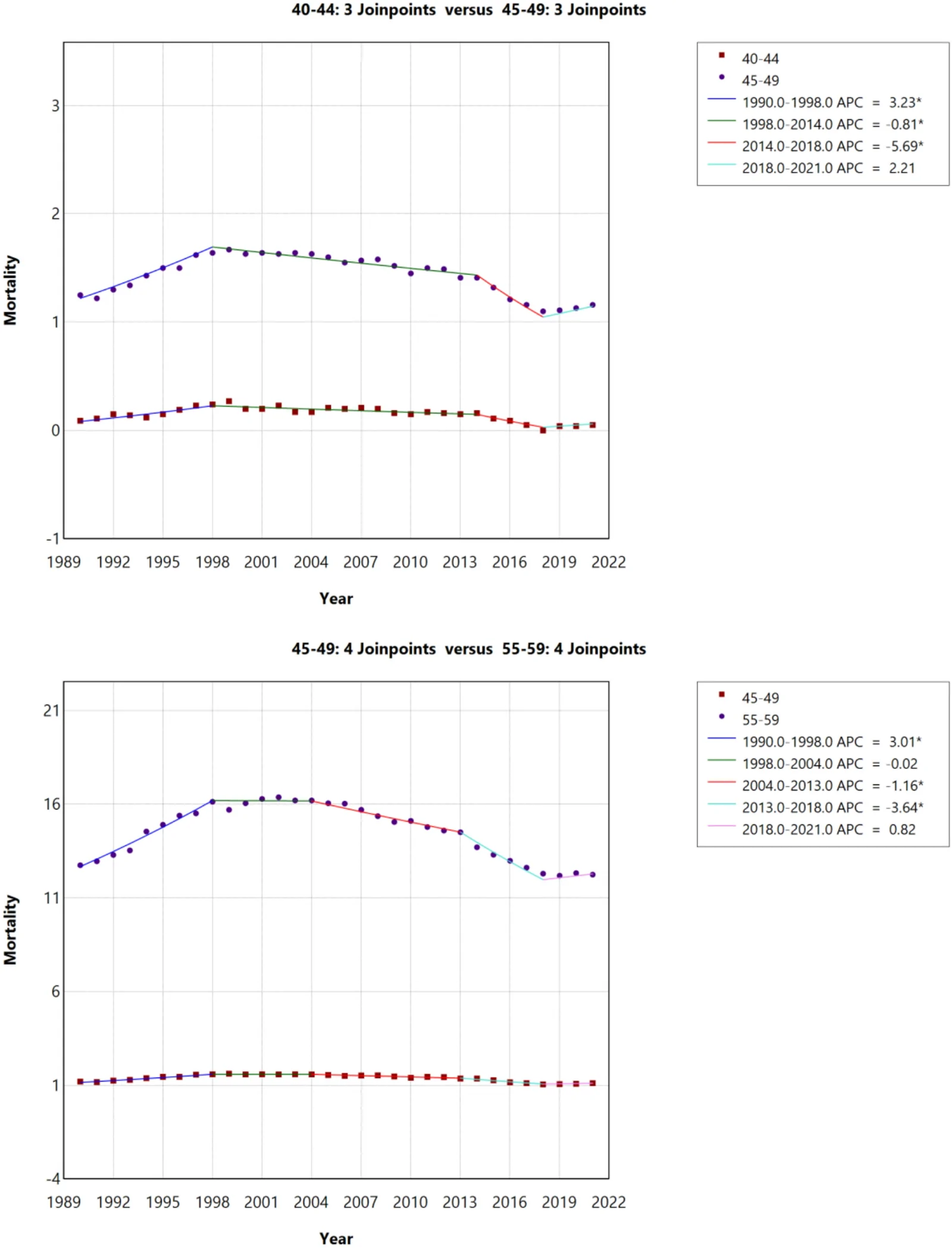
Pairwise comparison of prostate cancer mortality trends (age groups: 40–44, 45–49, and 55–59 years) demonstrating parallel trajectories from 1990 to 2021. Joinpoint regression analysis identifies significant inflection points (joinpoints) and corresponding annual percent change (APC) values for each trend segment. The parallel patterns suggest similar temporal dynamics across these age groups. Data derived from joinpoint regression (*Indicates that the Annual Percent Change (APC) is significantly different = *p* < 0.05)

In terms of mortality rates and age group relationships, parallelism was observed between the 45–49 and 40–44 age groups (3 joinpoints; *p* = 0.18), as well as between 45–49 and 55–59 age groups (4 joinpoints; *p* = 0.21). However, the AAPCs for both comparisons were non-significant, with −0.14% (95% CI −0.54 to 0.25; *p* = 0.47) and −0.09% (95% CI −0.35 to 0.16; *p* = 0.46), respectively. This demonstrates no significant difference in the average annual percent change between these groups over the entire period. No parallelism or significant AAPCs were observed in other pairwise age group combinations for mortality rates (Fig. 8).

**Fig. 9:**
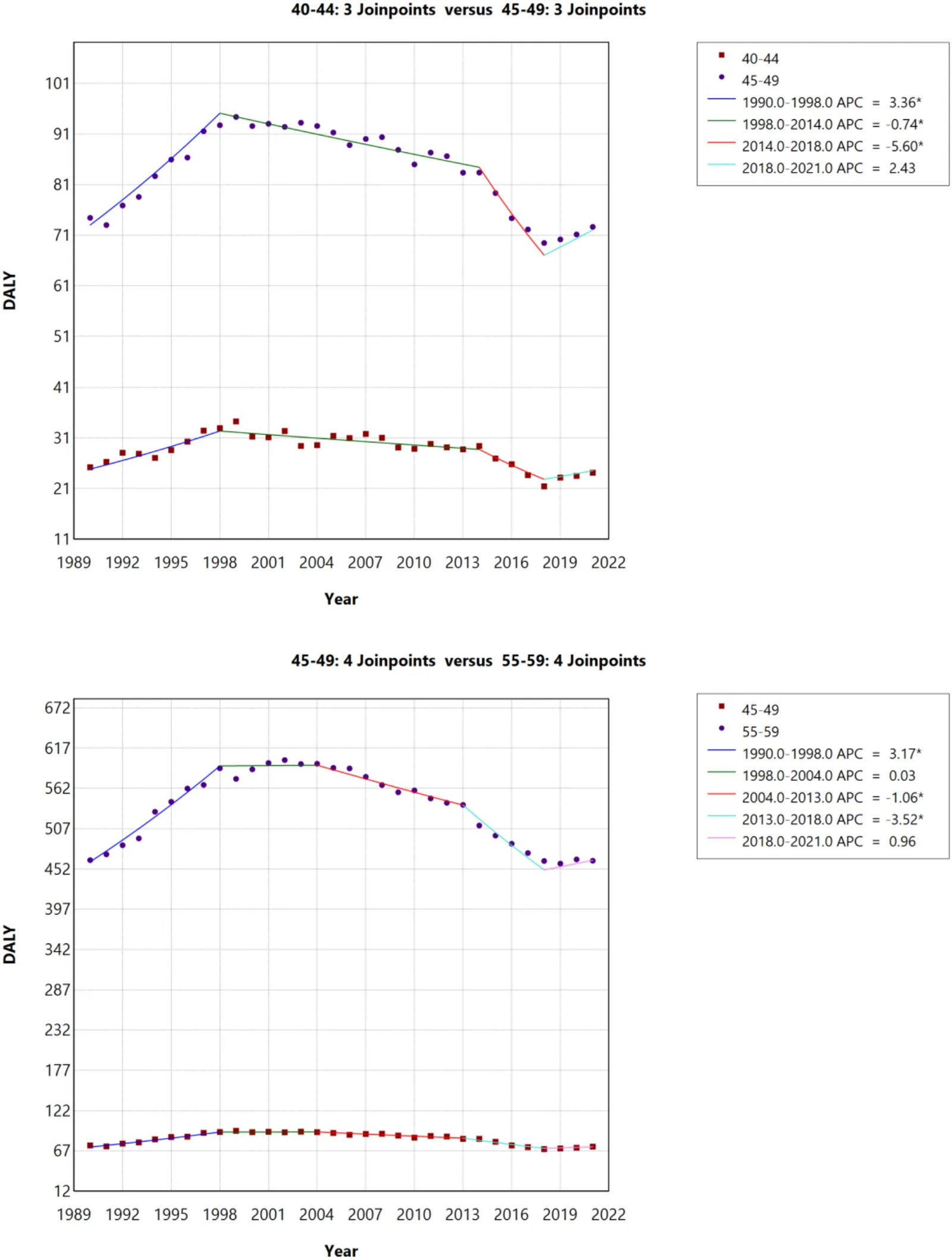
Pairwise comparison of prostate cancer DALY trends (age groups: 40–44, 45–49, and 55–59 years) demonstrating parallel trajectories from 1990 to 2021. Joinpoint regression analysis identifies significant inflection points (joinpoints) and corresponding annual percent change (APC) values for each trend segment. The parallel patterns suggest similar temporal dynamics across these age groups. Data derived from joinpoint regression (*Indicates that the Annual Percent Change (APC) is significantly different = *p* < 0.05)

Concerning DALY rates, the same pattern observed in other age groups was present. Specifically, parallelism was identified between the 45–49 and 40–44 age groups (3 joinpoints; *p* = 0.13) and between 45–49 and 55–59 age groups (4 joinpoints; *p* = 0.20), with no significant AAPCs (Average Annual Percent Changes) for either combination. No parallelism or significant AAPCs were observed in other pairwise age group comparisons for DALY rates (Fig. 9).

## Discussion

The findings of this study reinforce growing concerns about prostate cancer, a condition now established as a major public health challenge. The absolute increase in prevalence and disease burden observed in the analyzed data underscores critical healthcare system challenges, particularly regarding early diagnosis, treatment access, and overcoming regional and age-related disparities. In addressing Hypothesis 1— which posits epidemiological rate variations across macroregions and between age groups—we note a mortality stabilization trend among younger age groups, with low rates and minimal regional variation. This aligns with prostate cancer’s low incidence in individuals under 50 years. In intermediate and older age groups, we observe increasing mortality with advancing age and a peak-then-decline pattern in the South and Southeast regions, indicating positive impacts of early diagnosis and treatment. Regions with weaker healthcare infrastructure (notably North and Northeast) exhibit irregular patterns or sustain elevated mortality levels for longer durations. The study by Santos Filho et al. [25] corroborates our findings, even though using Mortality Information System (SIM) data, the authors emphasize that socioeconomic and educational inequalities among Brazilian regions likely exerted significant influence on observed prostate cancer mortality differentials.

Placing our findings within the broader context of the GBD 2021 study [17] is essential for understanding their significance. The global analysis demonstrated that non-communicable diseases, including prostate cancer, continue to contribute a substantial and growing burden, with age-standardized DALY rates for conditions like diabetes increasing by 14.0% between 2010 and 2021 [17]. This global trend aligns with our observation of increasing prostate cancer incidence and prevalence in Brazil, particularly in regions undergoing rapid demographic aging. However, the GBD 2021 study also underscores that national-level estimates often mask profound subnational heterogeneity [17]. Our study makes a critical contribution by quantifying this heterogeneity within Brazil. While GBD provides the high-quality data infrastructure, our work advances the literature in three specific ways; (1) Regional granularity: we demonstrate that the national trends for prostate cancer are composed of starkly contrasting regional patterns, such as declining mortality in the South and Southeast versus increasing mortality in the Northeast a divergence that would be lost in a national average; (2) Age-specific trend analysis: Through joinpoint regression, we identify precise inflection points in incidence trends across 5-year age cohorts, linking them to policy shifts like the 2012 and 2018 updates to prostate cancer screening guidelines; (3) Policy-oriented translation: We explicitly connect our epidemiological findings to Brazil’s unique public health context, including disparities in healthcare infrastructure, the structure of the Unified Health System (SUS), and the need for regionally tailored interventions. In doing so, we provide a model for how subnational GBD data can be leveraged to inform equitable health policy in large, federated countries.

Analysis of prostate cancer prevalence between 1990 and 2021 reveals substantial growth in age groups over 50 years, particularly in the South and Southeast regions, where rates remain consistently higher. This pattern likely reflects greater access to screening, early diagnosis, and prolonged survival. Conversely, the North and Northeast regions exhibit lower prevalence, though with an upward trajectory, potentially linked to progressive improvements in healthcare coverage alongside historical limitations in health service access, directly impacting disease detection. Previous studies corroborate these findings. As noted by Santos Filho et al. [25], elevated prostate cancer prevalence in the South and Southeast correlates with higher Human Development Index (HDI) scores, broader population-based screening coverage, and superior healthcare infrastructure. In contrast, recent increases in the North and Northeast partly reflect service expansion and enhanced data recording, despite persistent challenges in care accessibility and oncological equity.

Assessment of prostate cancer DALY trends in Brazil reveals minimal disease burden in younger age groups, consistent with the expected low incidence of prostatic neoplasia in this population. States in the Southeast and South regions exhibited the highest DALY values among older age groups, likely associated with greater post-diagnosis survival and improved access to early detection and treatment. On the other hand, the North and Northeast regions maintained lower DALY levels, potentially reflecting underreporting, limitations in accessing oncological diagnostics and treatment, and earlier mortality—which reduces disease duration and consequently years lived with disability. Brazil demonstrated stabilized or slightly decreasing DALY burden over the past three decades according to a Latin American and Mexican study, with Haiti, Dominican Republic, and Cuba showing the highest regional DALY burdens. These patterns may correlate with ethnic differences, biological factors, and socioeconomic status, while variable access to healthcare systems, health promotion activities, and systematic prostate cancer screening also exert distinct regional impacts nationwide [26]. Consistent with the trends observed in prostate cancer incidence data in Brazil, the projections align closely with mortality trends, with the North and Northeast regions exhibiting the highest incidence rates during the study period across most age groups over 40 years.

Support for Hypothesis 2, which explores the association between age groups and the prostate cancer burden, reveals striking epidemiological patterns. The study demonstrates that incidence, prevalence, and mortality increase progressively with age, peaking among individuals over 65 particularly those aged 75–89. This trend is consistent with established literature identifying aging as the primary risk factor, attributed to cumulative exposure to genetic mutations and hormonal changes [27]. For instance, incidence reached its highest level in the 75–84 age group, with 512.50 cases per 100,000 men in the South region, reflecting the region’s higher life expectancy. However, a slight decline was observed in the 90 + age group, potentially due to reduced population size and underreporting in this demographic [19].

The DALY burden, in turn, exhibited a distinct profile, with greatest impact in the 50–64 age group. This discrepancy reflects the societal weight of premature mortality and disability among economically active individuals. As noted by Luizaga et al. [12], years of life lost (YLL) and years lived with disability (YLD) in this population amplify the disease’s socioeconomic cost. In the Northeast region, for instance, DALYs among men aged 50–64 years showed annual increases of up to 0.85%, indicating deficiencies in early diagnosis access and effective treatments. These findings underscore the need for targeted policies toward younger adults, such as educational campaigns and selective screening—strategies long advocated by the Brazilian Society of Urology [4].

Analysis of non-linear temporal trends under Hypothesis 3 revealed three distinct phases through Joinpoint regression. The initial phase (1990–2010) featured substantial incidence increases, driven by expanded PSA-based screening implementation. This period aligned with aggressive early detection guidelines, exemplified by American Cancer Society [28] recommendations, which precipitated overdiagnosis of indolent tumors as evidenced in PLCO and ERSPC trials [5]. The subsequent phase (2012 onward) demonstrated incidence rate stabilization or decline, particularly in Southeast and South regions, following clinical guideline revisions by the U.S. Preventive Services Task Force [29]. These revisions established shared physician–patient decision-making as the prevailing standard, which remains current practice.

Mortality, conversely, demonstrated progressive reduction from 2000 onward, with annual declines reaching 0.70% in the Southeast. This decrease is associated with therapeutic advances notably conformational radiotherapy and second-generation hormone therapy which improved survival rates [19]. However, the Northeast exhibited opposing trends, showing significant increases in 65–69 age group (AAPC: + 0.76%), reflecting healthcare service quality disparities. Prevalence, in turn, rose consistently across all regions, reaching 2,619.50 cases per 100,000 men in the South (70–74 age group). This growth not only indicates extended survival but also highlights disease chronicity and population aging—factors emphasized by Zhou et al. [30] in global analyses.

Joinpoint graph analysis reveals distinct regional dynamics. For mortality, the South region exhibited significant declines in groups such as 70–74-year-olds (AAPC: −0.64%), whereas the Northeast showed persistent increases, most notably + 0.76% among 65–69-year-olds. These divergences suggest technological advances and treatment access proved more effective in economically developed regions, while structurally disadvantaged areas continue facing systemic barriers. Regarding incidence, the Northeast led increases across nearly all age groups (e.g., + 2.57% in 55–59 age group), attributable partly to recent detection improvements but also to risk factors including sedentary lifestyles and genetic predisposition in populations of African descent [2].

Pairwise comparisons of age groups revealed complex patterns, characterized by parallel temporal trajectories in prostate cancer incidence, prevalence, mortality, and DALY trends between 1990 and 2021. Significant APC values within specific trend segments suggest analogous temporal dynamics across the analyzed age cohorts. For instance, the 45–49 and 55–59 age groups exhibited parallel trajectories in incidence and prevalence, indicating that screening policies similarly impacted these demographics. In contrast, the absence of parallelism in other combinations (e.g., 55–59 vs. 65–69 years) suggests the influence of contextual variables, such as demographic shifts or changes in healthcare coverage. These findings underscore the critical importance of age-stratified analyses for guiding targeted interventions.

### Limitations

Methodological limitations prominently include the reliance on secondary data from the Global Burden of Disease (GBD) study, which may underestimate the disease burden in regions with fragile health systems, such as Brazil’s North and Northeast. As emphasized by the Institute for Health Metrics and Evaluation [13], the GBD’s statistical redistribution of “garbage codes” (ill-defined causes of death) can introduce biases in areas affected by chronic underreporting. Furthermore, macroregional aggregation obscures critical intraregional disparities—states such as Amazonas (North) and Bahia (Northeast) exhibit distinct epidemiological profiles that warrant individualized assessment.

Equally significant are practical constraints stemming from the impact of COVID-19 on 2020–2021 data, where pandemic-related disruptions to screening and treatment likely led to artificially reduced incidence and inflated short-term mortality, thereby distorting long-term trend interpretations, as noted by Yabroff et al. [31]. Compounding these challenges, the absence of comorbidity data (examples, diabetes, obesity) hinders a comprehensive analysis of disease progression determinants, which are essential for effective prevention strategies.

#### Comparative analysis of age cohorts in Brazil

Joinpoint Regression parallelism analysis revealed whether temporal trends across age groups shared statistically similar structural patterns. Examining prostate cancer incidence trends from 1990 to 2021 demonstrated curve similarity throughout this period. These findings indicate that middle-aged groups (45–59 age group) exhibit not only parallel trajectories but also sustained and homogeneous growth patterns in prostate cancer incidence, likely reflecting structural shifts in healthcare policy, increased disease awareness, and expanded access to screening tests such as PSA. Globally, a study of 43 populations observed that in countries such as the United States and Australia, the 1990 s introduction and dissemination of PSA testing precipitated sharp prostate cancer incidence increases, particularly among younger men. South Korea witnessed an approximate fourfold rise in prostate cancer incidence between 2006 and 2020, accompanied by an average annual 1.08% increase in PSA testing rates. This surge proved especially pronounced among men in their fifties, aligning with our findings [31, 32].

In contrast, older age groups exhibited non-parallel patterns compared to younger cohorts, indicating distinct temporal trajectories potentially linked to biological factors, evolving clinical practices, or demographic shifts among elderly populations. Analysis of US data (1995–2012) revealed declining localized/regional prostate cancer incidence in men aged 70 years or older, while advanced-stage disease rates increased in men aged 50 to 69 years from 2004 onward. Concurrently in China (1990–2017), individuals aged 50 to 79 years demonstrated substantially greater prostate cancer incidence increases relative to younger groups, with age effects showing pronounced upward trajectories emerging from age 40 onward, confirming divergent trend patterns in advanced age demographics [33, 34].

Prostate cancer prevalence trends during the study period revealed a parallel pattern between the 45–49 and 55–59-year age groups. The failure to reject parallelism suggests both cohorts experienced similar contextual influences, including evolving screening protocols, expanded PSA testing access, and diagnostic criteria refinements over time. These shared patterns reinforce how early detection strategies comparably impact middle-aged demographics, despite differing prevalence levels attributable to natural disease progression with aging. PSA screening within these age brackets significantly shapes prevalence trends, evidenced by a Swedish study comparing regularly screened men aged 50 to 54 years against a non-screened control group. After 17 years, the screened cohort demonstrated 2.56 times higher prostate cancer diagnosis incidence alongside significantly reduced disease-specific mortality relative to controls [35].

Analysis of prostate cancer mortality parallelism from 1990 to 2021 identified parallel trends between the 40–44 and 45–49 age groups, as well as between the 45–49 and 55–59 age cohorts. This similar temporal variation occurred despite non-significant changes in mortality rates, indicating overall stability. In contrast, other age groups exhibited divergent (non-parallel) trends, though they also showed no statistically significant shifts in mortality rates. A study conducted in the state of São Paulo (2000–2015) reported significant reductions in prostate cancer mortality specifically among individuals aged 50–59. Although it did not directly assess the 40–44 and 45–49 age groups, its findings suggest that adjacent age brackets may have experienced similar downward mortality trends over time. Meanwhile, research from a medium-sized city in Northeastern Brazil (1996–2015) identified rising prostate cancer incidence until 2007, followed by declining incidence and stable mortality—reflected in non-significant AAPCs across all age groups, in line with our statistical observations. Taken together, these studies support the presence of parallel mortality trends with temporal stability among the 40–44, 45–49, and 55–59 age groups, while other cohorts demonstrated distinct, though similarly stable, patterns [12, 36].

DALY rates demonstrated patterns consistent with other epidemiological metrics across age brackets. Current scientific literature lacks studies with comparable age-stratified detail for Brazil. The most relevant analysis—a 1990–2019 Latin American and Mexican study of prostate cancer burden across 20 countries including Brazil—revealed that most DALYs (91.7%) derived from years of life lost (YLLs), with substantial burden among men aged 55 years or older. Nevertheless, this research omitted granular age bracket trend analysis. Meanwhile, a global cancer burden study estimated prostate cancer accounted for 6.3 million DALYs in 2015: 82% attributable to YLLs and 18% to years lived with disability (YLDs). While providing comprehensive perspectives, neither study detailed specific age bracket trends or parallelism assessments [17, 26].

## Implications

These findings necessitate multifaceted interventions. In less developed regions such as the North and Northeast, urgent investments are required in diagnostic infrastructure including expanded PSA testing capacity and healthcare workforce training. Younger adults (50–64 years) should be the focus of targeted awareness campaigns addressing early symptoms and the importance of routine screenings, in line with INCA guidelines [1].Meanwhile, older populations require policies centered on quality of life, ensuring access to palliative care and multidisciplinary support. Ultimately, standardizing nationwide epidemiological registries with granular data on tumor staging and treatment modalities would enable more precise, actionable analyses to address these systemic challenges.

## Conclusions and perspectives

Given the growing burden posed by urological conditions, Brazil must enhance its public health policies to address the increasing demand for effective diagnosis, treatment, and prevention. It is essential for the country to invest in comprehensive strategies, including expanding access to specialized services, training healthcare professionals, promoting public education campaigns, and supporting scientific research in urology. Additionally, collaboration with international initiatives can facilitate the adoption of best practices and innovative technologies, ultimately contributing to a reduced disease burden and improved quality of life for the Brazilian population.

## Supplementary Information

Below is the link to the electronic supplementary material.

Supplementary file1 (TIFF 12657 KB)—Annualized change in mortality rates (per 100,000 men), 50-74 years, for prostate cancer across Brazilian macro-regions, 1990–2021. Green tones represent increases, red tones decrease. The map was created using data from the Global Burden of Disease Study 2021, with regional boundaries obtained from the Brazilian Institute of Geography and Statistics (IBGE) using geobr package in R

Supplementary file2 (TIFF 12657 KB)—Annualized change in prevalence rates (per 100,000 men), 50-74 years, for prostate cancer across Brazilian macro-regions, 1990–2021. Green tones represent increases, red tones decrease. The map was created using data from the Global Burden of Disease Study 2021, with regional boundaries obtained from the Brazilian Institute of Geography and Statistics (IBGE) using geobr package in R

Supplementary file3 (TIFF 12657 KB)—Annualized change in DALY rates (per 100,000 men), 50-74 years, for prostate cancer across Brazilian macro-regions, 1990–2021. Green tones represent increases, red tones decrease. The map was created using data from the Global Burden of Disease Study 2021, with regional boundaries obtained from the Brazilian Institute of Geography and Statistics (IBGE) using geobr package in R

Supplementary file4 (TIFF 12657 KB)—Annualized change in incidence rates (per 100,000 men), 50-74 years, for prostate cancer across Brazilian macro-regions, 1990–2021. Green tones represent increases, red tones decrease. The map was created using data from the Global Burden of Disease Study 2021, with regional boundaries obtained from the Brazilian Institute of Geography and Statistics (IBGE) using geobr package in R

Supplementary file5 (DOCX 35 KB)

## Data Availability

All data will be made available upon request to the authors.
