# Supplementary material for: Global burden of prostate cancer (1990–2021) from Brazil: trends in disability-adjusted life years, incidence, mortality, and prevalence
Source: Cancer Causes Control. 2026 Aug 19;37(9):147. doi: 10.1007/s10552-026-02232-2 (PMC13490226; doi:10.1007/s10552-026-02232-2)
Supplement: Supplementary file 1 [file 10552_2026_2232_MOESM5_ESM.docx]

**Table S1 -** Prostate Cancer Mortality Trends (1990–2021) by Region and Age Groups: Average Annual Percent Change (AAPC) with 95% Confidence Intervals (CI) derived from Joinpoint Regression Analysis. Trends are stratified by geographic region and age cohorts, with joinpoints indicating significant inflection points.

| Cohort (Region/Age – Joinpoints) | AAPC | Lower CI | Upper CI | P-Value |
| --- | --- | --- | --- | --- |
| South / 40-44 - 4 Joinpoints | -0.66 | -1.91 | 0.60 | 0.30 |
| Southeast / 40-44 - 4 Joinpoints | -0.51 | -1.93 | 0.93 | 0.48 |
| Central-West / 40-44 - 4 Joinpoints | -0.44 | -1.46 | 0.58 | 0.40 |
| North / 40-44 - 3 Joinpoints | 0.10 | -0.66 | 0.87 | 0.79 |
| Northeast / 40-44 - 6 Joinpoints | 0.07 | -1.03 | 1.19 | 0.90 |
| South / 45-49 - 4 Joinpoints | -0.58 | -1.25 | 0.09 | 0.09 |
| Southeast / 45-49 - 3 Joinpoints | -0.31 | -0.87 | 0.25 | 0.27 |
| Central-West / 45-49 - 6 Joinpoints | -0.46 | -1.36 | 0.45 | 0.32 |
| North / 45-49 - 5 Joinpoints | 0.46 | -0.69 | 1.63 | 0.44 |
| Northeast / 45-49 - 4 Joinpoints | 0.19 | -0.29 | 0.67 | 0.44 |
| South / 50-54 - 2 Joinpoints | -1.08* | -1.42 | -0.75 | < 0.001 |
| Southeast / 50-54 - 5 Joinpoints | -0.33 | -1.22 | 0.57 | 0.47 |
| Central-West / 50-54 - 5 Joinpoints | -0.39 | -1.52 | 0.76 | 0.504 |
| North / 50-54 - 6 Joinpoints | 0.11 | -0.73 | 0.95 | 0.80 |
| Northeast / 50-54 - 3 Joinpoints | 0.47* | 0.15 | 0.80 | 0.004 |
| South / 55-59 - 5 Joinpoints | -0.63* | -1.18 | -0.09 | 0.02 |
| Southeast / 55-59 - 5 Joinpoints | -0.31 | -0.73 | 0.10 | 0.13 |
| Central-West / 55-59 - 6 Joinpoints | -0.20 | -1.47 | 1.09 | 0.76 |
| North / 55-59 - 3 Joinpoints | 0.24 | -0.77 | 1.26 | 0.64 |
| Northeast / 55-59 - 6 Joinpoints | 0.69* | 0.16 | 1.23 | 0.01 |
| South / 60-64 - 4 Joinpoints | -0.48 | -0.98 | 0.02 | 0.06 |
| Southeast / 60-64 - 4 Joinpoints | -0.49* | -0.85 | -0.14 | 0.006 |
| Central-West / 60-64 - 5 Joinpoints | -0.27 | -1.21 | 0.67 | 0.57 |
| North / 60-64 - 3 Joinpoints | 0.58 | -0.01 | 1.17 | 0.05 |
| Northeast / 60-64 - 4 Joinpoints | 0.63* | 0.30 | 0.97 | < 0.001 |
| South / 65-69 - 3 Joinpoints | -0.64* | -1.07 | -0.22 | 0.003 |
| Southeast / 65-69 - 5 Joinpoints | -0.34 | -0.73 | 0.05 | 0.08 |
| Central-West / 65-69 - 3 Joinpoints | -0.42 | -1.04 | 0.21 | 0.19 |
| North / 65-69 - 2 Joinpoints | 0.77* | 0.26 | 1.28 | 0.003 |
| Northeast / 65-69 - 2 Joinpoints | 0.76* | 0.46 | 1.07 | < 0.001 |
| South / 70-74 - 4 Joinpoints | -0.42* | -0.82 | -0.03 | 0.03 |
| Southeast / 70-74 - 6 Joinpoints | -0.64* | -1.06 | -0.23 | 0.002 |
| Central-West / 70-74 - 3 Joinpoints | -0.49 | -1.22 | 0.25 | 0.20 |
| North / 70-74 - 3 Joinpoints | 0.68* | 0.10 | 1.27 | 0.02 |
| Northeast / 70-74 - 6 Joinpoints | 0.44 | -0.05 | 0.93 | 0.08 |
| South / 75-79 - 5 Joinpoints | -0.41 | -0.95 | 0.14 | 0.15 |
| Southeast / 75-79 - 5 Joinpoints | -0.51* | -0.95 | -0.08 | 0.02 |
| Central-West / 75-79 - 3 Joinpoints | -0.33 | -0.83 | 0.18 | 0.21 |
| North / 75-79 - 6 Joinpoints | 0.47 | -0.82 | 1.78 | 0.48 |
| Northeast / 75-79 - 5 Joinpoints | 0.67* | 0.39 | 0.96 | < 0.001 |
| South / 80-84 - 3 Joinpoints | -0.11 | -0.46 | 0.24 | 0.54 |
| Southeast / 80-84 - 4 Joinpoints | -0.70* | -0.97 | -0.45 | < 0.001 |
| Central-West / 80-84 - 3 Joinpoints | 0.29 | -0.24 | 0.82 | 0.29 |
| North / 80-84 - 4 Joinpoints | 0.79 | -0.17 | 1.76 | 0.11 |
| Northeast / 80-84 - 5 Joinpoints | 0.39 | -0.10 | 0.87 | 0.12 |
| South / 85-89 - 4 Joinpoints | 0.64* | 0.01 | 1.29 | 0.047 |
| Southeast / 85-89 - 4 Joinpoints | -0.16 | -0.56 | 0.25 | 0.45 |
| Central-West / 85-89 - 5 Joinpoints | 0.66 | -0.34 | 1.66 | 0.20 |
| North / 85-89 - 5 Joinpoints | 0.38 | -0.80 | 1.56 | 0.53 |
| Northeast / 85-89 - 2 Joinpoints | 0.81* | 0.46 | 1.18 | < 0.001 |
| South / 90-94 - 4 Joinpoints | 1.75* | 0.88 | 2.64 | < 0.001 |
| Southeast / 90-94 - 2 Joinpoints | -0.07 | -0.39 | 0.26 | 0.69 |
| Central-West / 90-94 - 3 Joinpoints | 0.69 | -0.43 | 1.83 | 0.23 |
| North / 90-94 - 4 Joinpoints | -0.02 | -1.27 | 1.25 | 0.98 |
| Northeast / 90-94 - 4 Joinpoints | 1.15* | 0.49 | 1.82 | < 0.001 |
| South / 95+ - 3 Joinpoints | -0.03 | -1.07 | 1.02 | 0.96 |
| Southeast / 95+ - 2 Joinpoints | -1.58* | -2.12 | -1.04 | < 0.001 |
| Central-West / 95+ - 3 Joinpoints | 0.63 | -0.89 | 2.17 | 0.42 |
| North / 95+ - 4 Joinpoints | -0.64 | -1.63 | 0.36 | 0.21 |
| Northeast / 95+ - 5 Joinpoints | 0.79* | 0.09 | 1.50 | 0.03 |

*Indicates that the AAPC is significantly

**Table S2 -** Prostate Cancer Prevalence Trends (1990–2021) by Region and Age Groups: Average Annual Percent Change (AAPC) with 95% Confidence Intervals (CI) derived from Joinpoint Regression Analysis. Trends are stratified by geographic region and age cohorts, with joinpoints indicating significant inflection points.

| Cohort (Region/Age – Joinpoints) | AAPC | Lower CI | Upper CI | P-Value |
| --- | --- | --- | --- | --- |
| South / 40-44 - 4 Joinpoints | 1.42* | 0.42 | 2.44 | 0.005 |
| Southeast / 40-44 - 4 Joinpoints | 1.97* | 0.90 | 3.06 | < 0.001 |
| Central-West / 40-44 - 4 Joinpoints | 1.80* | 0.72 | 2.89 | 0.001 |
| North / 40-44 - 6 Joinpoints | 2.10* | 1.22 | 3.00 | < 0.001 |
| Northeast / 40-44 - 4 Joinpoints | 2.10* | 1.38 | 2.83 | < 0.001 |
| South / 45-49 - 4 Joinpoints | 1.53* | 0.90 | 2.18 | < 0.001 |
| Southeast / 45-49 - 3 Joinpoints | 2.24* | 1.70 | 2.79 | < 0.001 |
| Central-West / 45-49 - 4 Joinpoints | 1.84* | 1.11 | 2.58 | < 0.001 |
| North / 45-49 - 5 Joinpoints | 2.37* | 1.34 | 3.42 | < 0.001 |
| Northeast / 45-49 - 6 Joinpoints | 2.21* | 1.58 | 2.86 | < 0.001 |
| South / 50-54 - 3 Joinpoints | 1.32* | 0.56 | 2.09 | < 0.001 |
| Southeast / 50-54 - 5 Joinpoints | 2.24* | 1.32 | 3.17 | < 0.001 |
| Central-West / 50-54 - 3 Joinpoints | 2.02* | 1.43 | 2.63 | < 0.001 |
| North / 50-54 - 3 Joinpoints | 2.12* | 1.63 | 2.62 | < 0.001 |
| Northeast / 50-54 - 6 Joinpoints | 2.55* | 2.05 | 3.06 | < 0.001 |
| South / 55-59 - 5 Joinpoints | 1.59* | 1.13 | 2.06 | < 0.001 |
| Southeast / 55-59 - 4 Joinpoints | 2.24* | 1.79 | 2.70 | < 0.001 |
| Central-West / 55-59 - 6 Joinpoints | 2.18* | 0.50 | 3.89 | 0.01 |
| North / 55-59 - 2 Joinpoints | 2.26* | 1.81 | 2.72 | < 0.001 |
| Northeast / 55-59 - 6 Joinpoints | 2.85* | 2.32 | 3.39 | < 0.001 |
| South / 60-64 - 4 Joinpoints | 1.53* | 0.99 | 2.09 | < 0.001 |
| Southeast / 60-64 - 4 Joinpoints | 1.98* | 1.63 | 2.34 | < 0.001 |
| Central-West / 60-64 - 6 Joinpoints | 2.01* | 1.05 | 2.98 | < 0.001 |
| North / 60-64 - 4 Joinpoints | 2.5* | 1.74 | 3.28 | < 0.001 |
| Northeast / 60-64 - 3 Joinpoints | 2.59* | 2.22 | 2.98 | < 0.001 |
| South / 65-69 - 6 Joinpoints | 1.5* | 1.14 | 1.87 | < 0.001 |
| Southeast / 65-69 - 5 Joinpoints | 2.15* | 1.68 | 2.63 | < 0.001 |
| Central-West / 65-69 - 3 Joinpoints | 1.87* | 1.37 | 2.38 | < 0.001 |
| North / 65-69 - 3 Joinpoints | 2.62* | 1.80 | 3.46 | < 0.001 |
| Northeast / 65-69 - 3 Joinpoints | 2.74* | 2.30 | 3.17 | < 0.001 |
| South / 70-74 - 3 Joinpoints | 1.68* | 1.46 | 1.91 | < 0.001 |
| Southeast / 70-74 - 5 Joinpoints | 1.78* | 1.50 | 2.08 | < 0.001 |
| Central-West / 70-74 - 6 Joinpoints | 1.8* | 0.98 | 2.64 | < 0.001 |
| North / 70-74 - 4 Joinpoints | 2.59* | 1.74 | 3.46 | < 0.001 |
| Northeast / 70-74 - 6 Joinpoints | 2.39* | 1.95 | 2.84 | < 0.001 |
| South / 75-79 - 4 Joinpoints | 1.81* | 1.48 | 2.14 | < 0.001 |
| Southeast / 75-79 - 4 Joinpoints | 1.99* | 1.66 | 2.33 | < 0.001 |
| Central-West / 75-79 - 4 Joinpoints | 2.04* | 1.36 | 2.73 | < 0.001 |
| North / 75-79 - 3 Joinpoints | 2.36* | 1.49 | 3.25 | < 0.001 |
| Northeast / 75-79 - 6 Joinpoints | 2.57* | 2.25 | 2.90 | < 0.001 |
| South / 80-84 - 6 Joinpoints | 2.01* | 1.44 | 2.60 | < 0.001 |
| Southeast / 80-84 - 5 Joinpoints | 1.81* | 1.50 | 2.12 | < 0.001 |
| Central-West / 80-84 - 4 Joinpoints | 2.64* | 1.97 | 3.32 | < 0.001 |
| North / 80-84 - 4 Joinpoints | 2.54* | 1.57 | 3.53 | < 0.001 |
| Northeast / 80-84 - 6 Joinpoints | 2.13* | 1.68 | 2.58 | < 0.001 |
| South / 85-89 - 5 Joinpoints | 2.11* | 1.53 | 2.70 | < 0.001 |
| Southeast / 85-89 - 4 Joinpoints | 1.58* | 1.31 | 1.86 | < 0.001 |
| Central-West / 85-89 - 5 Joinpoints | 2.19* | 1.38 | 3.01 | < 0.001 |
| North / 85-89 - 5 Joinpoints | 1.65* | 0.85 | 2.47 | < 0.001 |
| Northeast / 85-89 - 4 Joinpoints | 1.80* | 1.38 | 2.24 | < 0.001 |
| South / 90-94 - 4 Joinpoints | 1.55* | 1.01 | 2.10 | < 0.001 |
| Southeast / 90-94 - 2 Joinpoints | 0.62* | 0.43 | 0.82 | < 0.001 |
| Central-West / 90-94 - 3 Joinpoints | 1.14* | 0.67 | 1.62 | < 0.001 |
| North / 90-94 - 3 Joinpoints | 0.42 | -0.07 | 0.91 | 0.09 |
| Northeast / 90-94 - 5 Joinpoints | 1.03* | 0.50 | 1.58 | < 0.001 |
| South / 95+ - 3 Joinpoints | 0.57* | 0.21 | 0.95 | 0.002 |
| Southeast / 95+ - 2 Joinpoints | -0.21 | -0.46 | 0.04 | 0.09 |
| Central-West / 95+ - 2 Joinpoints | 0.49* | 0.13 | 0.87 | 0.009 |
| North / 95+ - 4 Joinpoints | -0.07 | -0.60 | 0.46 | 0.79 |
| Northeast / 95+ - 5 Joinpoints | 0.68* | 0.31 | 1.07 | < 0.001 |

*Indicates that the AAPC is significantly

**Table S3 -** Prostate Cancer DALYs Trends (1990–2021) by Region and Age Groups: Average Annual Percent Change (AAPC) with 95% Confidence Intervals (CI) derived from Joinpoint Regression Analysis. Trends are stratified by geographic region and age cohorts, with joinpoints indicating significant inflection points.

| Cohort (Region/Age – Joinpoints) | AAPC | Lower CI | Upper CI | P-Value |
| --- | --- | --- | --- | --- |
| South / 40-44 - 4 Joinpoints | -0.57 | -1.73 | 0.60 | 0.34 |
| Southeast / 40-44 - 4 Joinpoints | -0.35 | -1.59 | 0.90 | 0.58 |
| Central-West / 40-44 - 4 Joinpoints | -0.37 | -1.36 | 0.62 | 0.46 |
| North / 40-44 - 3 Joinpoints | 0.17 | -0.58 | 0.92 | 0.66 |
| Northeast / 40-44 - 6 Joinpoints | 0.15 | -0.96 | 1.27 | 0.80 |
| South / 45-49 - 4 Joinpoints | -0.48 | -1.12 | 0.16 | 0.14 |
| Southeast / 45-49 - 3 Joinpoints | -0.20 | -0.78 | 0.38 | 0.49 |
| Central-West / 45-49 - 6 Joinpoints | -0.36 | -1.27 | 0.55 | 0.44 |
| North / 45-49 - 5 Joinpoints | 0.53 | -0.67 | 1.74 | 0.39 |
| Northeast / 45-49 - 4 Joinpoints | 0.23 | -0.23 | 0.69 | 0.32 |
| South / 50-54 - 2 Joinpoints | -0.95* | -1.29 | -0.62 | < 0.001 |
| Southeast / 50-54 - 5 Joinpoints | -0.20 | -1.10 | 0.70 | 0.66 |
| Central-West / 50-54 - 5 Joinpoints | -0.27 | -1.41 | 0.88 | 0.64 |
| North / 50-54 - 6 Joinpoints | 0.19 | -0.62 | 1.02 | 0.64 |
| Northeast / 50-54 - 3 Joinpoints | 0.56* | 0.25 | 0.88 | < 0.001 |
| South / 55-59 - 5 Joinpoints | -0.51 | -1.06 | 0.04 | 0.07 |
| Southeast / 55-59 - 5 Joinpoints | -0.18 | -0.59 | 0.23 | 0.39 |
| Central-West / 55-59 - 6 Joinpoints | -0.08 | -1.35 | 1.21 | 0.90 |
| North / 55-59 - 3 Joinpoints | 0.33 | -0.67 | 1.34 | 0.52 |
| Northeast / 55-59 - 6 Joinpoints | 0.79* | 0.25 | 1.34 | 0.004 |
| South / 60-64 - 4 Joinpoints | -0.37 | -0.89 | 0.14 | 0.15 |
| Southeast / 60-64 - 4 Joinpoints | -0.37* | -0.73 | -0.02 | 0.04 |
| Central-West / 60-64 - 5 Joinpoints | -0.16 | -1.10 | 0.80 | 0.75 |
| North / 60-64 - 3 Joinpoints | 0.67* | 0.09 | 1.25 | 0.02 |
| Northeast / 60-64 - 4 Joinpoints | 0.73* | 0.40 | 1.06 | < 0.001 |
| South / 65-69 - 3 Joinpoints | -0.53* | -0.98 | -0.09 | 0.02 |
| Southeast / 65-69 - 5 Joinpoints | -0.22 | -0.60 | 0.15 | 0.24 |
| Central-West / 65-69 - 3 Joinpoints | -0.33 | -0.95 | 0.28 | 0.29 |
| North / 65-69 - 2 Joinpoints | 0.84* | 0.35 | 1.35 | < 0.001 |
| Northeast / 65-69 - 2 Joinpoints | 0.85* | 0.56 | 1.15 | < 0.001 |
| South / 70-74 - 3 Joinpoints | -0.40* | -0.62 | -0.19 | < 0.001 |
| Southeast / 70-74 - 3 Joinpoints | -0.58* | -0.78 | -0.40 | < 0.001 |
| Central-West / 70-74 - 3 Joinpoints | -0.39 | -1.14 | 0.36 | 0.31 |
| North / 70-74 - 3 Joinpoints | 0.76* | 0.19 | 1.34 | 0.009 |
| Northeast / 70-74 - 6 Joinpoints | 0.52* | 0.03 | 1.02 | 0.039 |
| South / 75-79 - 5 Joinpoints | -0.33 | -0.87 | 0.22 | 0.24 |
| Southeast / 75-79 - 5 Joinpoints | -0.43* | -0.87 | 0.00 | 0.049 |
| Central-West / 75-79 - 3 Joinpoints | -0.25 | -0.77 | 0.27 | 0.35 |
| North / 75-79 - 6 Joinpoints | 0.54 | -0.75 | 1.84 | 0.41 |
| Northeast / 75-79 - 4 Joinpoints | 0.78* | 0.55 | 1.02 | < 0.001 |
| South / 80-84 - 3 Joinpoints | -0.06 | -0.41 | 0.28 | 0.71 |
| Southeast / 80-84 - 4 Joinpoints | -0.65* | -0.92 | -0.39 | < 0.001 |
| Central-West / 80-84 - 3 Joinpoints | 0.32 | -0.22 | 0.87 | 0.25 |
| North / 80-84 - 4 Joinpoints | 0.82 | -0.15 | 1.79 | 0.10 |
| Northeast / 80-84 - 3 Joinpoints | 0.43* | 0.05 | 0.81 | 0.03 |
| South / 85-89 - 4 Joinpoints | 0.66* | 0.01 | 1.31 | 0.045 |
| Southeast / 85-89 - 5 Joinpoints | -0.15 | -0.76 | 0.47 | 0.63 |
| Central-West / 85-89 - 5 Joinpoints | 0.66 | -0.34 | 1.67 | 0.20 |
| North / 85-89 - 5 Joinpoints | 0.38 | -0.80 | 1.57 | 0.53 |
| Northeast / 85-89 - 2 Joinpoints | 0.82* | 0.46 | 1.19 | < 0.001 |
| South / 90-94 - 4 Joinpoints | 1.75* | 0.87 | 2.65 | < 0.001 |
| Southeast / 90-94 - 2 Joinpoints | -0.07 | -0.39 | 0.26 | 0.68 |
| Central-West / 90-94 - 3 Joinpoints | 0.68 | -0.43 | 1.81 | 0.23 |
| North / 90-94 - 4 Joinpoints | -0.02 | -1.26 | 1.24 | 0.98 |
| Northeast / 90-94 - 4 Joinpoints | 1.15* | 0.49 | 1.82 | < 0.001 |
| South / 95+ - 3 Joinpoints | -0.20 | -1.32 | 0.93 | 0.73 |
| Southeast / 95+ - 2 Joinpoints | -1.76* | -2.32 | -1.20 | < 0.001 |
| Central-West / 95+ - 3 Joinpoints | 0.85 | -1.49 | 3.25 | 0.48 |
| North / 95+ - 4 Joinpoints | -0.73 | -1.73 | 0.29 | 0.16 |
| Northeast / 95+ - 5 Joinpoints | 0.82* | 0.11 | 1.55 | 0.02 |

*Indicates that the AAPC is significantly

**Table S4 -** Prostate Cancer Incidence Trends (1990–2021) by Region and Age Groups: Average Annual Percent Change (AAPC) with 95% Confidence Intervals (CI) derived from Joinpoint Regression Analysis. Trends are stratified by geographic region and age cohorts, with joinpoints indicating significant inflection points.

| Cohort (Region/Age – Joinpoints) | AAPC | Lower CI | Upper CI | P-Value |
| --- | --- | --- | --- | --- |
| South / 40-44 - 4 Joinpoints | 1.32* | 0.29 | 2.37 | 0.01 |
| Southeast / 40-44 - 4 Joinpoints | 1.79* | 0.73 | 2.85 | < 0.001 |
| Central-West / 40-44 - 4 Joinpoints | 1.64* | 0.49 | 2.8 | 0.004 |
| North / 40-44 - 3 Joinpoints | 1.82* | 0.90 | 2.74 | < 0.001 |
| Northeast / 40-44 - 4 Joinpoints | 1.91* | 1.15 | 2.68 | < 0.001 |
| South / 45-49 - 4 Joinpoints | 1.37* | 0.73 | 2.01 | < 0.001 |
| Southeast / 45-49 - 3 Joinpoints | 1.99* | 1.43 | 2.55 | < 0.001 |
| Central-West / 45-49 - 4 Joinpoints | 1.61* | 0.86 | 2.37 | < 0.001 |
| North / 45-49 - 5 Joinpoints | 2.2* | 1.12 | 3.28 | < 0.001 |
| Northeast / 45-49 - 6 Joinpoints | 1.98* | 1.32 | 2.64 | < 0.001 |
| South / 50-54 - 2 Joinpoints | 1.00* | 0.66 | 1.34 | < 0.001 |
| Southeast / 50-54 - 5 Joinpoints | 1.95* | 1.10 | 2.82 | < 0.001 |
| Central-West / 50-54 - 3 Joinpoints | 1.77* | 1.16 | 2.37 | < 0.001 |
| North / 50-54 - 3 Joinpoints | 1.89* | 1.40 | 2.39 | < 0.001 |
| Northeast / 50-54 - 6 Joinpoints | 2.30* | 1.79 | 2.82 | < 0.001 |
| South / 55-59 - 5 Joinpoints | 1.34* | 0.87 | 1.81 | < 0.001 |
| Southeast / 55-59 - 4 Joinpoints | 1.91* | 1.46 | 2.36 | < 0.001 |
| Central-West / 55-59 - 6 Joinpoints | 1.88* | 0.18 | 3.60 | 0.02 |
| North / 55-59 - 2 Joinpoints | 2.03* | 1.54 | 2.52 | < 0.001 |
| Northeast / 55-59 - 6 Joinpoints | 2.57* | 2.03 | 3.12 | < 0.001 |
| South / 60-64 - 4 Joinpoints | 1.18* | 0.61 | 1.75 | < 0.001 |
| Southeast / 60-64 - 4 Joinpoints | 1.55* | 1.18 | 1.91 | < 0.001 |
| Central-West / 60-64 - 6 Joinpoints | 1.59* | 0.60 | 2.59 | 0.001 |
| North / 60-64 - 4 Joinpoints | 2.17* | 1.41 | 2.92 | < 0.001 |
| Northeast / 60-64 - 3 Joinpoints | 2.24* | 1.80 | 2.69 | < 0.001 |
| South / 65-69 - 3 Joinpoints | 1.08* | 0.56 | 1.60 | < 0.001 |
| Southeast / 65-69 - 4 Joinpoints | 1.55* | 1.23 | 1.88 | < 0.001 |
| Central-West / 65-69 - 3 Joinpoints | 1.32* | 0.81 | 1.84 | < 0.001 |
| North / 65-69 - 2 Joinpoints | 2.21* | 1.71 | 2.71 | < 0.001 |
| Northeast / 65-69 - 3 Joinpoints | 2.26* | 1.83 | 2.69 | < 0.001 |
| South / 70-74 - 3 Joinpoints | 0.98* | 0.71 | 1.24 | < 0.001 |
| Southeast / 70-74 - 5 Joinpoints | 0.89* | 0.55 | 1.24 | < 0.001 |
| Central-West / 70-74 - 6 Joinpoints | 1.00* | 0.15 | 1.86 | 0.02 |
| North / 70-74 - 4 Joinpoints | 1.94* | 1.08 | 2.81 | < 0.001 |
| Northeast / 70-74 - 6 Joinpoints | 1.69* | 1.20 | 2.18 | < 0.001 |
| South / 75-79 - 5 Joinpoints | 0.71* | 0.18 | 1.25 | 0.008 |
| Southeast / 75-79 - 4 Joinpoints | 0.67* | 0.34 | 1.00 | < 0.001 |
| Central-West / 75-79 - 5 Joinpoints | 0.80 | -0.02 | 1.64 | 0.05 |
| North / 75-79 - 5 Joinpoints | 1.36* | 0.27 | 2.47 | 0.013 |
| Northeast / 75-79 - 6 Joinpoints | 1.60* | 1.15 | 2.05 | < 0.001 |
| South / 80-84 - 2 Joinpoints | 0.61* | 0.36 | 0.87 | < 0.001 |
| Southeast / 80-84 - 4 Joinpoints | 0.10 | -0.18 | 0.39 | 0.46 |
| Central-West / 80-84 - 4 Joinpoints | 1.06* | 0.34 | 1.79 | 0.003 |
| North / 80-84 - 4 Joinpoints | 1.33* | 0.36 | 2.31 | 0.006 |
| Northeast / 80-84 - 4 Joinpoints | 0.96* | 0.46 | 1.46 | < 0.001 |
| South / 85-89 - 4 Joinpoints | 1.05* | 0.40 | 1.71 | 0.001 |
| Southeast / 85-89 - 5 Joinpoints | 0.28 | -0.35 | 0.92 | 0.38 |
| Central-West / 85-89 - 5 Joinpoints | 1.07* | 0.07 | 2.08 | 0.03 |
| North / 85-89 - 5 Joinpoints | 0.66 | -0.50 | 1.85 | 0.26 |
| Northeast / 85-89 - 3 Joinpoints | 1.15* | 0.77 | 1.53 | < 0.001 |
| South / 90-94 - 4 Joinpoints | 1.84* | 0.97 | 2.73 | < 0.001 |
| Southeast / 90-94 - 2 Joinpoints | 0.02 | -0.30 | 0.36 | 0.86 |
| Central-West / 90-94 - 3 Joinpoints | 0.78 | -0.36 | 1.93 | 0.18 |
| North / 90-94 - 4 Joinpoints | 0.04 | -1.20 | 1.30 | 0.94 |
| Northeast / 90-94 - 4 Joinpoints | 1.21* | 0.54 | 1.89 | < 0.001 |
| South / 95+ - 3 Joinpoints | 0.06 | -0.96 | 1.10 | 0.9 |
| Southeast / 95+ - 2 Joinpoints | -1.48* | -2.07 | -0.89 | < 0.001 |
| Central-West / 95+ - 3 Joinpoints | 0.70 | -0.79 | 2.21 | 0.35 |
| North / 95+ - 4 Joinpoints | -0.57 | -1.57 | 0.43 | 0.26 |
| Northeast / 95+ - 5 Joinpoints | 0.86* | 0.15 | 1.57 | 0.01 |

*Indicates that the AAPC is significantly
